# Modification of Boc-Protected CAN508 via Acylation and Suzuki-Miyaura Coupling

**DOI:** 10.3390/molecules23010149

**Published:** 2018-01-12

**Authors:** Martin Pisár, Eva Schütznerová, Filip Hančík, Igor Popa, Zdeněk Trávníček, Petr Cankař

**Affiliations:** 1 Department of Organic Chemistry, Faculty of Science, Palacký University, 17. listopadu 1192/12, 771 46 Olomouc, Czech Republic; martin.pisar01@upol.cz (M.P.); Filda.Hancik@seznam.cz (F.H.); 2 Institute of Molecular and Translation Medicine, Faculty of Medicine, Palacký University, Hněvotínská 5, 779 00 Olomouc, Czech Republic; eva.schutznerova@upol.cz; 3 Department of Inorganic Chemistry, Faculty of Science, Palacký University, 17. listopadu 1192/12, 771 46 Olomouc, Czech Republic; igor.popa@upol.cz (I.P.); zdenek.travnicek@upol.cz (Z.T.)

**Keywords:** pyrazole, Boc-protection, Suzuki-Miyaura reaction, acylation, CDK inhibitor, XPhos Pd G2

## Abstract

The cyclin-dependent kinase inhibitor, CAN508, was protected with di-*tert*-butyl dicarbonate to access the amino-benzoylated pyrazoles. The bromo derivatives were further arylated by Suzuki-Miyaura coupling using the XPhos Pd G2 pre-catalyst. The coupling reaction provided generally the *para*-substituted benzoylpyrazoles in the higher yields than the *meta*-substituted ones. The Boc groups were only utilized as directing functionalities for the benzoylation step and were hydrolyzed under conditions of Suzuki-Miyaura coupling, which allowed for elimination of the additional deprotection step.

## 1. Introduction

**Figure 1:**
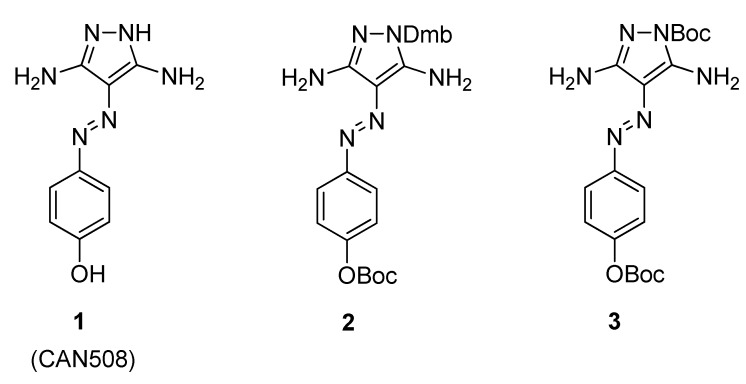
Derivatives of pyrazole **1** protected at the endocyclic pyrazole nitrogen and hydroxyl group.

Previously, we reported an acylation study [1] to substitute regioselectively the amino group of cyclin-dependent kinase (CDK) [2,3] inhibitor **1** (CAN508) [4]. Pyrazole **2** was protected with the 2,4-dimethoxybenzyl group (Dmb) and with Boc (Figure 1). The Dmb was selectively and fully introduced at the pyrazole endocyclic nitrogen using 2,4-dimethoxybenzylhydrazine. The additional *ortho*-methoxy group in Dmb enabled milder deprotection conditions in comparison to the described *p*-methoxybenzyl group (PMB) [1,5,6,7,8].

Despite the methodology using Dmb is unequivocally regioselective, there is still a disadvantage associated with the introduction of Dmb via a 3-step synthesis. To simplify the protecting methodology, it is desirable to prepare pyrazole **3** (Figure 1).

Protecting groups are often used as auxiliary tools in the synthesis of biological active compounds [9]. For example, the Boc protection of the endocyclic pyrazole nitrogen was used in the synthesis of TBK1 inhibitor Tozasertib [10], selective CDK4 inhibitors based on the pyrazol-3-yl urea scaffold [11], CDK2 inhibitor PHA-533533 [12], or selective SGK1 inhibitors [13]. 

The regioselective protection of pyrazoles is inherently complicated due to tautomerism of a pyrazole heterocyclic system [14]. Moreover, if a pyrazole system is in conjugation with peripheral functional groups, the tautomeric system is more complex, which results in even more difficult regioselective protection.

The Boc-protection of a 3(5)-aminopyrazole system represents an illustrative example, where both endocyclic nitrogens and the amino groups can be protected concurrently [15,16]. The protection usually led to a mixture of mono- and di-substituted regio-isomers, which are difficult to purify. To avoid that, several methods were developed: (i) the acylation of aminopyrazoles with 2 equiv. of acyl reagents followed with saponification of the acyl group attached at the endocyclic pyrazole nitrogen [17]; (ii) the oxidation-reduction sequence of the amino group including Boc-protection of the endocyclic pyrazole nitrogen [18]; and (iii) the direct Boc-protection under alkaline conditions [16].

**Scheme 1:**
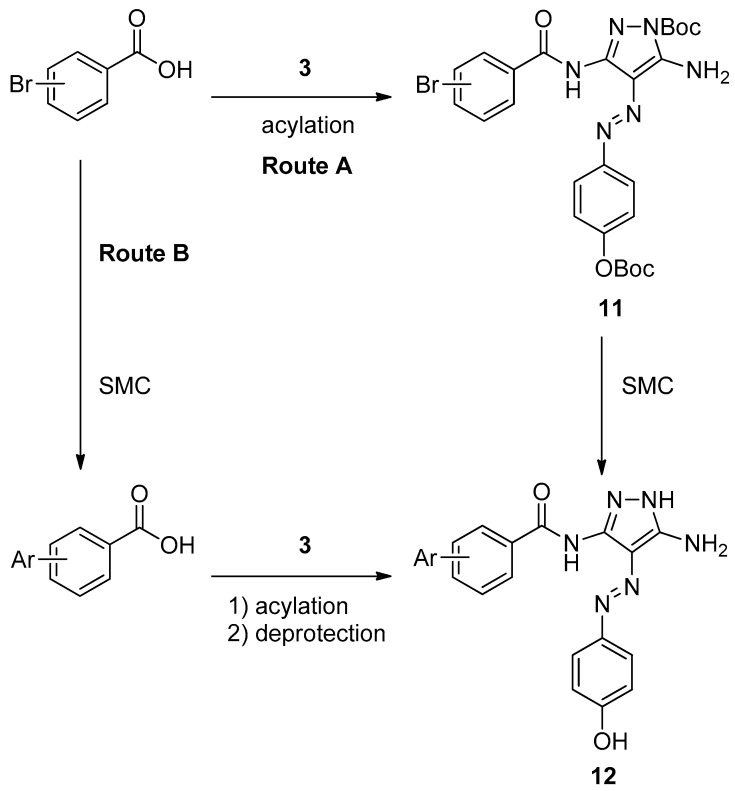
Possible synthetic routes leading to pyrazoles **12**.

Furthermore, since the X-ray crystallography of the CAN508-CDK2 complex indicated a possible extension of structure optimization to improve the CDK inhibition activity [4], we initiated a concept to use pyrazole **3** as a starting intermediate for synthetic **Route A** to give directly unprotected pyrazoles **12** (Scheme 1).

**Route A** can be potentially an alternative to conventional **Route B** and offer other synthetic strategies in drug development of pyrazole derivatives with an unprotected endocyclic nitrogen. The synthetic sequence (**Route A**) employing acylation before the Suzuki-Miyaura coupling (SMC) [19] exposes the catalyst in the SMC reaction to more functionalized reactants/products with a potentially higher coordination activity.

The classical catalysts with triphenylphosphine ligands are not robust and are frequently inhibited or decomposed, when such unprotected functionalized pyrazoles are used. The sterically-bulky phosphines were also inefficient [20,21]. Consequently, the SMC reaction has to precede the acylation (**Route B**) or the pyrazole endocyclic nitrogen is substituted to enable a coupling reaction with reasonable yields [22].

In the last decade, the development of ligands and their use as pre-catalysts significantly broaden the scope of the SMC coupling with various nitrogen-containing heterocyclic systems [23,24,25]. Recently, we have taken an advantage of these scope extensions and reported studies with the challenging aminopyrazole substrates [20,21,26], which encourage us to attempt to synthesize pyrazoles **12** via **Route A**.

In our endeavours to find more efficient CDK inhibitors based on the structure of pyrazole **1** (CAN508) [14,27,28,29], we decided to use the Boc-protection of pyrazole **1**, subsequent benzoylation of the Boc-protected pyrazole **3**, and the final arylation under SMC conditions to further explore the robustness of a catalyst originated from the XPhos Pd G2 pre-catalyst.

## 2. Results and Discussion

### 2.1. Boc-Protection and Benzoylation

**Scheme 2:**
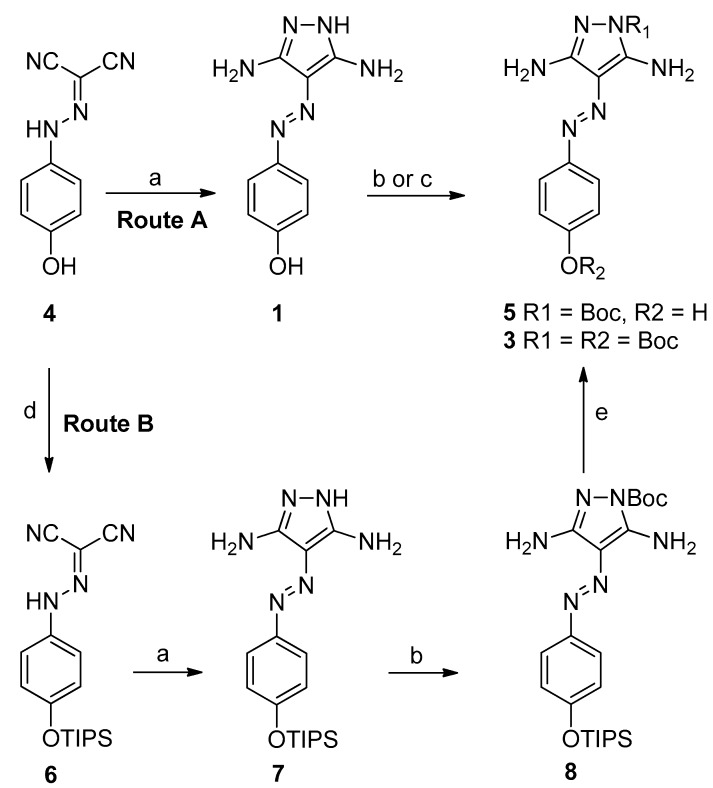
Two independent routes leading to Boc-protected pyrazoles **5** and **3**. Reagents and conditions: (**a**) N_2_H_4_.H_2_O (1.5 equiv.), MeOH, reflux, 4 h, for **7**: 97%; (**b**) Boc_2_O (1.05 equiv.), anhydrous DMF, r.t., 18 h, for **5**: 99%, for **8**: 77%; (**c**) for **3**: Boc_2_O (2 equiv.), pyridine, r.t., 18 h, 94%; (**d**) anhydrous DMF in dichloromethane (DCM) (10%), imidazole (1.1 equiv.), TIPS-Cl (1.1 equiv.), r.t., 18h 59%; (**e**) TBAF (1 equiv.), MeOH, r.t., 18 h (91%).

We found that the reaction of pyrazole **1** with acyl chlorides proceeded at the endocyclic nitrogen [1]. To redirect the acylation reaction toward the amino group it is necessary to protect the endocyclic pyrazole nitrogen and the hydroxyl group. Preliminary experiments with di-*tert*-butyl dicarbonate provided mono-protected pyrazole **5** and di-protected derivative **3**, and showed a significant role of a base (Scheme 2, **Route A**). While the protection with di-*tert*-butyl dicarbonate in anhydrous dimethylformamide (DMF) gave pure mono-Boc protected derivative **5**, a reaction in pyridine resulted in a mixture of mono- and di-Boc protected derivatives **5** and **3**. Another equivalent of di-*tert*-butyl dicarbonate provided solely pyrazole **3**.

To verify that the first mono-Boc protection took place at the diaminopyrazole moiety, we chose an alternative indirect synthetic approach involving an additional protecting group (Scheme 2, **Route B**). At first, hydrazone **4** was protected with a triisopropylsilyl (TIPS) group to give compound **6**, which subsequently underwent cyclyzation with hydrazine to yield pyrazole **7**. Then, TIPS-protected pyrazole **7** was treated with di-*tert*-butyl dicarbonate to obtain derivative **8**. The final selective unmasking of the hydroxyl group with TBAF resulted in the target pyrazole **5**.

**Figure 2:**
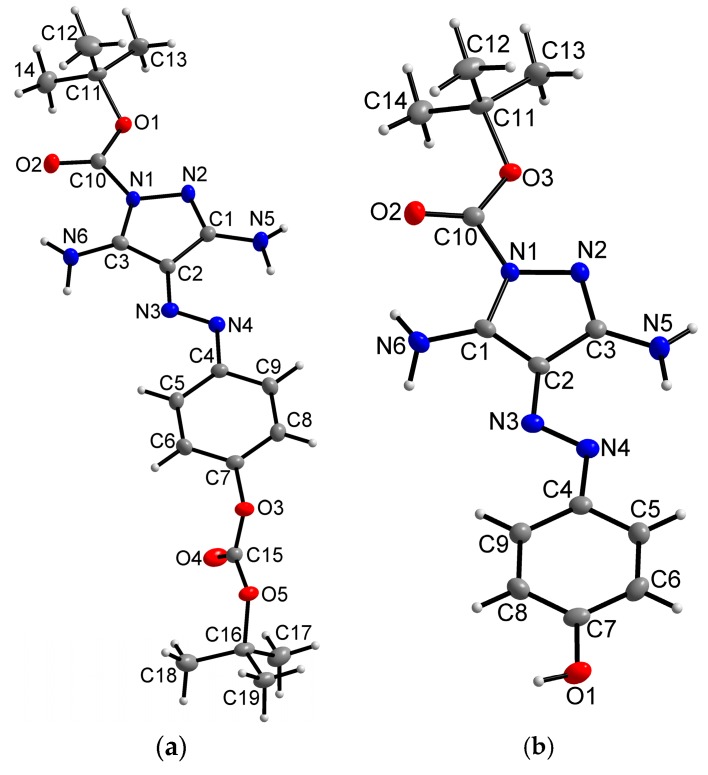
Molecular structures: (**a**) pyrazole **3** (CCDC 1453321) and (**b**) pyrazole **5** (CCDC 1453320), together with the atom numbering schemes. The thermal ellipsoids of the non-hydrogen atoms are drawn at the 50% probability level.

While the alternative synthesis of pyrazole **5** confirmed the position of the Boc group at the diaminopyrazole moiety, the single crystal X-ray analysis of **5** unequivocally determined the exact position of the Boc group at the pyrazole endocyclic nitrogen (Figure 2). X-ray crystallography also confirmed the position of the second Boc group at the hydroxyl in pyrazole **3**. Selected bond lengths and angles of **3** and **5** can be found in the Supplementary file. Moreover, the X-ray analysis also revealed that the crystal structures of both compounds are stabilized by hydrogen bonds of the N–H···N, N–H···O and O–H···N types, situating the individual molecules into supramolecular 1D-polymeric chains (see Figures S1 and S2 in the Supplementary File). Each of the supramolecular chains is connected through a variety of the non-covalent contacts (e.g., N–H···N, C–H···O and/or C–H···N) with two neighboring ones, thus forming a supramolecular-layered structure (see Figures S3 and S4 in the Supplementary File).

**Scheme 3:**
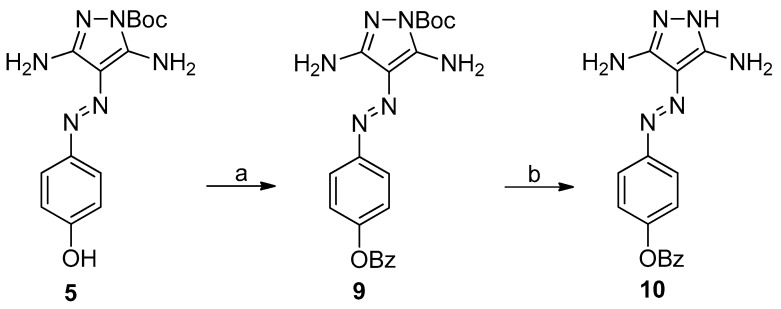
Benzoylation of pyrazole **5**. Reagents and conditions: (**a**) BzCl (1.5 equiv.), triethylamine (1.5 equiv.), dry DMF, DCM, r.t., 18 h (47%); (**b**) 10% TFA in DCM, r.t., 1.5 h (81%).

To confirm the markedly preferred reactivity of the hydroxyl over the amino groups, we carried out benzoylation of pyrazole **5** with benzoyl chloride (Scheme 3). This reaction resulted in pyrazol **9**. Subsequent deprotection with diluted trifluoroacetic acid (TFA) provided benzoylated pyrazol **10**. The predominant reactivity of the hydroxyl group highlighted the necessity of its protection.

The acylation of pyrazole **3** with benzoyl chlorides provided amino-benzoylated pyrazoles **11a**–**c** (Table 1). Since benzoylated pyrazoles **11a** and **11b** were accompanied with an increased amount of impurities, the crude products were directly deprotected in the next step without isolation. The subsequent cleavage of both Boc groups was carried out initially under conventional conditions with diluted TFA to yield **12a** (Table 1, entry 1). In view of the following Suzuki-Miyaura coupling, tripotassium phosphate was additionally used to deprotect pyrazoles **11b**–**c** in the very good to excellent yields (Table 1, entries 2 and 3).

### 2.2. Suzuki-Miyaura Coupling

In order to extend the diversity of pharmacologically relevant amino benzoylated pyrazoles **12b**–**c**, we used our previously reported SMC conditions for dinitropyrazoles [26]. Preliminary deprotection experiments with tripotassium phosphate showed that already two thirds of **11c** were converted after 30 min into **12c** and after 1 h the cleavage was almost complete. The first coupling experiments with p-tolylboronic acid enabled for providing biphenyl **12d** in a very good yield (Table 2, entry 1), which indicated the robustness of the catalyst [30]. The comparable yields were obtained with benzene and 4-methoxybenzeneboronic acids (entries 2 and 3). The ortho-substitution did not affect the reactivity negatively; it was possible to isolate **12g** in the excellent yield (entry 4). However, the electron-withdrawing nitro group decreased the yield (entry 5). Electron-rich furan-3-yl and thiophen-3-yl boronic acids reacted with **11c** in the very good or excellent yields (entries 6 and 7).

Since the benzoylation of pyrazole **11b** was accompanied by the mono-Boc impurity, and it was found that the catalyst is already exposed to a substantial excess of unprotected pyrazoles **12b** and **12c** in the earliest stage of the coupling, we decided to perform the coupling reaction directly with unprotected pyrazole **12b**. Preliminary attempts showed a more difficult coupling reaction at the *meta* position. The HPLC analyses usually confirmed the presence of unreacted pyrazole **12b**, the desired coupling product, and other unknown impurities. The yields were considerably lower and only pyrazoles **12k**–**n** were possible to isolate (Table 3).

## 3. Experimental Section

### General

All starting materials are commercially available. Commercial reagents were used without purification. Melting points were determined on a Boetius stage and are uncorrected. Flash column chromatography was performed on silica gel (pore size 60 Å, 40–63 mm particle size). The LC/MS analyses were carried out on a UHPLC-MS system consisting of UHPLC chromatography Accela with photodiode array detector and triple quadrupole mass spectrometer TSQ Quantum Access (both Thermo Scientific, Santa Clara, CA, USA), using Nucleodur Gravity C18 column at 30 °C and flow rate of 800 μL/min (Kinetex, Phenomenex, 2.6 μm, 2.1 × 50 mm, Torrance, CA, USA). The mobile phase was (A) 0.01M ammonium acetate in water, and (B) acetonitrile, linearly programmed from 10% to 80% B over 2.5 min, kept for 1.5 min. The column was reequilibrated with a 10% of solution B for 1 min. The atmospheric-pressure chemical ionization (APCI) source operated at discharge current of 5 μA, vaporizer temperature of 400 °C, and capillary temperature of 200 °C. The HRMS analyses were carried out on HRMSdExactive (Orbitrap) MS, Thermo Scientific, Santa Clara, CA, USA. The ^1^H- and ^13^C-NMR spectra were measured in DMSO-*d*_6_ on a Bruker Avance 300 FT NMR spectrometer (Billerica, MA, USA) and on a Varian 400 MHz FT NMR spectrometer (Palo Alto, CA, USA). The single crystal X-ray data of 3 (CCDC 1453321) and 5 (CCDC 1453320) were obtained using an Xcalibur2 diffractometer equipped with a Sapphire2 CCD detector (Oxford Diffraction Ltd., Abingdon, UK), and with MoKα radiation (monochromator Enhance, Oxford Diffraction Ltd) and ω-scan technique at 120 K. Additional details regarding structure determinations, such as crystal data and structure refinements, selected bond lengths and angles of covalent as well as non-covalent contacts are summarized in the Supplementary File.

*Tert-butyl 3,5-diamino-4-((4-((tert-butoxycarbonyl)oxy)phenyl)diazenyl)-1H-pyrazole-1-carboxylate* (**3**): To a solution of pyrazole **1** (218 mg, 1.0 mmol) [4] in dry pyridine (6 mL), di-*tert*-butyl dicarbonate (0.460 mL, 2.0 mmol) diluted with dry pyridine (2 mL) was added dropwise at 2–5 °C. The reaction mixture was allowed to stir at room temperature for 18 h. After that, the solvent was removed under reduced pressure and the residue was diluted in methanol (6 mL). The solution was added dropwise to ice water (30 mL). The precipitate was filtered-off, washed with water, and dried in the air to give **3** as a yellow solid (392 mg, 94%); m.p. 148–150 °C; ^1^H-NMR (400 MHz, DMSO-*d*_6_) δ = 7.82 (br s, 2H), 7.80 (d, *J* = 8.8 Hz, 2H), 7.23 (d, *J* = 8.8, 2H), 6.15 (br s, 2H), 1.54 (s, 9H), 1.48 (s, 9H) ppm; ^13^C-NMR (101 MHz, DMSO-*d*_6_) δ = 27.7, 28.2, 83.7, 84.3, 113.6, 122.3, 122.3, 149.7, 150.1, 150.5, 150.6, 151.2, 151.6 ppm; HRMS (HESI, *m*/*z*) calcd. for C_19_H_26_N_6_O_5_ (418.45) [M + H]^+^ 419.2037, found 419.2037.

*Tert-butyl 3,5-diamino-4-((4-hydroxyphenyl)diazenyl)-1H-pyrazole-1-carboxylate* (**5**)*:*

*Reaction of unprotected pyrazole* **1** *with Boc-ahydride:* To a solution of pyrazole **1** (218 mg, 1.0 mmol) [4] in dry DMF (2 mL), di-*tert-*butyl dicarbonate (0.240 mL, 1.1 mmol) diluted with dry DMF (1 mL) was added dropwise at 2–5 °C. The reaction mixture was allowed to stir at room temperature for 18 h and then was added dropwise to ice water (15 mL). The precipitate was filtered-off, washed with water, and dried in the air to give **5** as a yellow solid (314 mg, 99%).

*TIPS deprotection of the hydroxyl group:* To a solution of TIPS-protected pyrazole **8** (200 mg, 0.42 mmol) in methanol (40 mL), tetrabutylammonium fluoride hydrate (TBAF; 110 mg, 0.42 mmol) was added. The reaction mixture was allowed to stir at room temperature for 18 h. After that the solvent was evaporated under reduced pressure and the residue was diluted in methanol (5 mL). The solution was added dropwise to ice water (25 mL). The precipitate was filtered-off, washed with water, and dried in the air to give 5. Yield 122 mg (91%) as a yellow solid; m.p. 114–116 °C; ^1^H-NMR (400 MHz, DMSO-*d*_6_) δ = 9.75 (br s, 1H), 7.63 (d, *J* = 8.8 Hz, 2H), 7.59 (br s, 2H), 6.79 (d, *J* = 8.8 Hz, 2H), 6.02 (br s, 2H), 1.53 (s, 9H) ppm; ^13^C-NMR (101 MHz, DMSO-*d*_6_) δ = 28.2, 84.0, 112.6, 115.9, 123.0, 145.4, 146.4, 150.7, 152.1, 158.6 ppm; HRMS (HESI, *m*/*z*) calcd. for C_14_H_18_N_6_O_3_ (318.33) [M + H]^+^ 319.1515, found 319.1515.

*(4-((Triisopropylsilyl)oxy)phenyl)carbonohydrazonoyl dicyanide (***6***)*: Hydrazone **4** (186 mg, 1.0 mmol) [1,4] was dissolved in a mixture of dry DCM (5 mL) and DMF (0.5 mL), then imidazole (75 mg, 1.1 mmol) and triisopropylsilyl chloride (TIPS-Cl; 235 μL, 1.1 mmol) were added under continuous stirring and cooling on an ice-bath. The reaction mixture was stirred at room temperature for 18 h. After that, the solvent was removed under reduced pressure and the residue was diluted in methanol (2 mL). The solution was added dropwise to ice water (10 mL). The precipitate was filtered-off, washed with water, and dried in the air. Crude hydrazone **6** was crystallized from methanol (3 mL) to give a yellow solid. Yield 202 mg (59%); m.p. 136–138 °C; ^1^H-NMR (300 MHz, DMSO-*d*_6_) δ = 7.37 (d, *J* = 9.0 Hz, 2H), 6.91 (d, *J* = 9.0 Hz, 2H), 1.16–1.30 (m, 3H), 1.04 (d, *J* = 7.1 Hz, 18H) ppm; ^13^C-NMR (75 MHz, DMSO-*d*_6_) δ = 12.0, 17.7, 82.9, 110.3, 114.7, 118.04, 120.39, 135.51, 153.66 ppm; HRMS (HESI, *m*/*z*) calcd. for C_18_H_26_N_4_OSi (342.51) [M − H]^−^ 341.1792, found 341.1796.

*4-((4-((Triisopropylsilyl)oxy)phenyl)diazenyl)-1H-pyrazole-3,5-diamine (***7***):* To a solution of hydrazone **6** (342 mg, 1.0 mmol) in methanol (15 mL), hydrazine hydrate (64%; 73 μL, 1.5 mmol) was added dropwise at room temperature. The solution was heated at 65 °C for 4 h. After cooling to room temperature, the solvent was evaporated under reduced pressure and the residue was diluted in methanol (2 mL). The solution was added dropwise to ice water (10 mL). The precipitate was filtered-off, washed with water, and dried in the air to give pyrazole **7** as a yellow solid. Yield 363 mg, 97%; m.p. 184–186 °C; ^1^H-NMR (400 MHz, DMSO-*d*_6_) δ = 10.63 (s, 1H), 7.56 (d, *J* = 8.8 Hz, 2H), 6.85 (d, *J* = 8.8 Hz, 2H), 6.18 (br s, 2H), 5.63 (br s, 2H), 1.23 (sxt, *J* = 7.5 Hz, 3H), 1.06 (s, 9H), 1.04 (s, 9H) ppm; ^13^C-NMR (101 MHz, DMSO-*d*_6_) δ = 2.5, 18.2, 114.0, 120.2, 122.2, 148.5, 154.9 ppm; HRMS (HESI, *m*/*z*) calcd. for C_18_H_30_N_6_OSi (374.23) [M + H]^+^ 375.2323, found 375.2325.

*Tert-butyl 3,5-diamino-4-((4-((triisopropylsilyl)oxy)-phenyl)diazenyl)-1H-pyrazole-1-carboxylate (***8**): To a solution of pyrazole **7** (374 mg, 1.0 mmol) in dry DMF (6 mL), di-*tert*-butyl dicarbonate (0.240 mL, 1.05 mmol) diluted with dry DMF (1 mL) was added dropwise at 2–5 °C. The reaction mixture was allowed to stir at room temperature for 18 h and then was added dropwise to ice water (30 mL). The precipitate was filtered-off, washed with water, and dried in the air to give **8** as a yellow solid. Yield 365 mg, 77%, m.p. 88–90 °C; ^1^H-NMR (400 MHz, DMSO-*d*_6_) δ = 7.69 (br s, 2H), 7.68 (d, *J* = 8.8 Hz, 2H), 6.89 (d, *J* = 8.8 z, 2H), 6.07 (br s, 2H), 1.53 (s, 9H), 1.24 (sxt, *J* = 7.5 Hz, 3H), 1.06 (s, 9H), 1.04 (s, 9H) ppm; ^13^C-NMR (101 MHz, DMSO-*d*_6_) δ = 12.5, 18.2, 28.2, 84.1, 113.0, 120.3, 122.9, 146.5, 148.0, 150.7, 152.1, 156.0 ppm; HRMS (HESI, *m*/*z*) calcd. for C_23_H_38_N_6_O_3_Si (474.28) [M + H]^+^ 475.2847, found 475.2851.

*Tert-butyl 3,5-diamino-4-((4-(benzoyloxy)phenyl)-diazenyl)-1H-pyrazole-1-carboxylate (***9***):* To a solution of pyrazole **5** (318 mg, 1.0 mmol) and triethylamine (209 μL, 1.5 mmol) in anhydrous DMF (10 mL), a solution of benzoyl chloride (175 μL, 1.5 mmol) diluted with DCM (3 mL) was added dropwise at 2–5 °C. The reaction mixture was allowed to stir at room temperature for 18 h. After that, the reaction mixture was added dropwise to ice water (50 mL). The precipitate was filtered-off, washed with water, and dried in the air to give **9** as a yellow solid. Yield 199 mg 47%; m.p. 188–190 °C; ^1^H-NMR (400 MHz, DMSO-*d*_6_) δ = 8.14 (d, *J* = 7.6 Hz, 2H); 7.88 (d, *J* = 8.8 Hz, 2H), 7.87 (br s, 2H), 7.74 (t, *J* = 7.5 Hz, 1H), 7.61 (t, *J* = 7.6 Hz, 2H), 7.35 (d, *J* = 8.8 Hz, 2H), 6.18 (br s, 2H), 1.54 (s, 9H) ppm; ^13^C-NMR (101 MHz, DMSO-*d*_6_) δ = 28.2, 84.3, 113.7, 122.4, 122.8, 129.4, 129.5, 130.3, 134.5, 144.0, 150.5, 150.7, 151.3, 151.4, 165.1 ppm; HRMS (HESI, *m*/*z*) calcd. for C_21_H_22_N_6_O_4_ (422.44) [M + H]^+^ 423.1775, found 423.1774.

*4-((3,5-Diamino-1H-pyrazol-4-yl)diazenyl)phenyl benzoate (***10***):* Boc-protected pyrazole **9** (0.5 mmol) was dissolved in 10% TFA/DCM (15 mL). The reaction mixture was allowed to stir at room temperature for 1 h. Then, the solvent was evaporated under reduced pressure. The residue was diluted with water (20 mL), pH was adjusted with ammonia to 10, and the suspension was allowed to stir at room temperature for 2 h. The precipitate was filtered-off, washed with water, and dried in the air to give **10** as a yellow solid. Yield 130 mg (81%); m.p. 196–198 °C; ^1^H-NMR (400 MHz, DMSO-*d*_6_) δ = 10.75 (br s, 1H), 8.14 (d, *J* = 7.9 Hz, 2H), 7.74 (d, *J* = 8.8 Hz, 2H), 7.74 (tt, *J* = 1.3, 7.1 Hz, 1H), 7.60 (t, *J* = 7.8 Hz, 2H), 7.29 (dd, *J* = 2.0, 8.8 Hz, 2H), 6.30 (br s, 2H), 5.87 (br s, 2H) ppm; ^13^C-NMR (101 MHz, DMSO-*d*_6_) δ = 114.7, 121.7, 122.6, 129.4, 130.0, 130.2, 134.5, 144.5, 149.5, 151.5, 152.0, 165.1 ppm; HRMS (HESI, *m*/*z*) calcd. for C_16_H_14_N_6_O_2_ (322.32) [M + H]^+^ 323.1251, found 323.1250.

*General procedure for* (**11**): To a solution of pyrazole **3** (3.8 g, 9.1 mmol) in pyridine (13 mL) the corresponding benzoyl chloride (9.1 mmol) was added in small portions under continuous stirring at 2–5 °C. Then, the reaction mixture was allowed to stir at room temperature for 3 h. After that, the solvent was evaporated under reduced pressure, the residue was diluted with methanol (15 mL), and a methanolic solution (or suspension) was added dropwise into ice water (50 mL). The precipitate was filtered-off, washed with water, and dried in the air to give **11**.

*Tert-butyl 5-amino-3-benzamido-4-((4-((tert-butoxycarbonyl)oxy)phenyl)diazenyl)-1H-pyrazole-1-carboxylate* (**11a**): The benzoylation was performed at the 1 mmol scale. Yield 481 mg as a yellow solid (a crude product contains impurities). Then, the product was directly used for the Boc-deprotection step (please see **12a**).

*Tert-butyl 5-amino-3-(3-bromobenzamido)-4-((4-((tert-butoxycarbonyl)oxy)phenyl)diazenyl)-1H-pyrazole-1-carboxylate* (**11b**): Yield 4.5 g (a crude product contains a mono-Boc protected pyrazole as an impurity). Then, the product was directly used for the Boc-deprotection step (please see **12b**).

*Tert-butyl 5-amino-3-(4-bromobenzamido)-4-((4-((tert-butoxycarbonyl)oxy)phenyl)diazenyl)-1H-pyrazole-1-carboxylate* (**11c**): Yield 4.6 g (84%) as a yellow solid; m.p. 128–132 °C; ^1^H-NMR (400 MHz,DMSO-*d*_6_) δ = 10.87 (s, 1H), 8.08 (br s, 2H), 7.94 (d, *J* = 8.3 Hz, 2H), 7.77 (d, *J* = 8.3 Hz, 2H), 7.70 (d, *J* = 8.8 Hz, 2H), 7.27 (d, *J* = 8.8 Hz, 2H), 1.60 (s, 9 H), 1.49 (s, 9H) ppm; ^13^C-NMR (101 MHz, DMSO-*d*_6_) δ = 164.9, 151.0, 150.7, 150.3, 149.5, 146.2, 143.6, 132.6, 131.7, 129.9, 126.0, 122.0, 115.7, 85.5, 83.4, 27.6, 27.2 ppm; HRMS (HESI, *m*/*z*) calcd. for C_26_H_29_BrN_6_O_6_ (601.46) [M − H]^−^ 601.1227, found 601.1220.

*N-(5-Amino-4-((4-hydroxyphenyl)diazenyl)-1H-pyrazol-3-yl)benzamide* (**12a**): Pyrazole **11a** (0.5 mmol) was dissolved in 10% TFA/DCM (15 mL). The reaction mixture was allowed to stir at room temperature for 2.5 h. The solvent was evaporated under reduced pressure. The residue was dissolved in water (20 mL) and pH was adjusted with aqueous ammonia to pH 10. The suspension was allowed to stir at room temperature for 2 h. The product **12a** was filtered-off, washed with water, and dried in the air. Yield 148 mg (92%) as a yellow solid. The compound was identical with the pyrazole published previously [1].

*N-(5-Amino-4-[(4-hydroxyphenyl)diazenyl)-1H-pyrazol-3-yl]-3-bromobenzamide* (**12b**): A crude product of Boc-protected pyrazole **11b** (1.0 mmol) and K_3_PO_4_ (3.0 mmol) was dissolved in a solution of 1,4-dioxane/water (5 mL, 4/1). The reaction mixture was vigorously stirred at 100 °C for 1.5 h, then cooled down to room temperature, neutralized with diluted HCl (1/5) to pH ≈ 7, and diluted with water (20 mL). The precipitate was filtered-off, washed with water, and dried in the air. Yield 321 mg (80%) as an orange solid; m.p. 249–252 °C; ^1^H-NMR (400 MHz, DMSO-*d*_6_) δ = 11.81 (br s, 1H), 10.63 (br s, 1H), 9.77 (br s, 1H), 8.14 (t, *J* = 1.8 Hz, 1H), 7.97 (d, *J* = 7.8 Hz, 1H), 7.82 (dd, *J* = 1.5, 7.8 Hz, 1H), 7.54 (d, *J* = 8.7 Hz, 2H), 7.53 (t, *J* = 7.8 Hz, 1H), 6.81 (d, *J* = 8.7 Hz, 2H), 6.60 (br s, 2H) ppm; ^13^C-NMR (101 MHz, DMSO-*d*_6_) δ = 115.5, 116.2, 121.9, 122.5, 126.8, 130.4, 130.9, 134.6, 136.1, 143.3, 145.9, 158.1, 164.1 ppm; HRMS (HESI, *m*/*z*) calcd. for C_16_H_13_BrN_6_O_2_ (401.22) [M − H]^−^ 399.0205, found 399.0201.

*N-(5-Amino-4-[(4-hydroxyphenyl)diazenyl)-1H-pyrazol-3-yl]-4-bromobenzamide* (**12c**): Prepared analogously as **12b**. Yield 381 mg (95%) as an orange solid; m.p. 268–273 °C; ^1^H-NMR (400 MHz, DMSO-*d*_6_) δ = 10.65 (br s, 3H), 7.99 (d, *J* = 8.7 Hz, 2H), 7.68 (d, *J* = 8.2 Hz, 2H), 7.52 (d, *J* = 9.2 Hz, 2H), 6.80 (d, *J* = 8.7 Hz, 2H), 6.28 (br s, 2H) ppm; ^13^C-NMR (101 MHz, DMSO-*d*_6_) δ = 115.5, 116.7, 122.1, 124.4, 130.0, 131.1, 135.7, 144.2, 146.1, 157.6, 166.4 ppm; HRMS (HESI, *m*/*z*) calcd. for C_16_H_13_BrN_6_O_2_ (401.22) [M − H]^−^ 399.0205, found 399.0917.

*General procedure of Suzuki-Miyaura coupling for* (**12d**–**n**): A mixture of pyrazole **11c** or **12b** (0.25 mmol), (hetero)arylboronic acid (0.5 mmol, 2 equiv.), and K_3_PO_4_ (212 mg, 1.0 mmol, 4 equiv.) in 1,4-dioxane/water (2.5 mL, 4/1) was bubbled with argon for 5 min in a 10 mL capped Supelco vial . Then, the XPhos Pd G2 pre-catalyst (10 mg, 0.0125 mmol, 5 mol %) was added. The reaction mixture was bubbled with argon again for 5 min, vigorously stirred at 100 °C for 24 h, and then was cooled down to room temperature, neutralized with diluted HCl (1/5) to pH ≈ 7, and diluted with water (10 mL). The precipitate was filtered-off, washed with water, and dried in the air. The crude product was purified on a silica gel column (EtOAc, then EtOAc/MeOH = 5/1).

*N-(5-Amino-4-((4-hydroxyphenyl)diazenyl)-1H-pyrazol-3-yl)-4′-methyl-[1,1′-biphenyl]-4-carboxamide* (**12d**): Yield 91 mg (88%) as an orange solid; m.p. 261–265 °C; ^1^H-NMR (400 MHz, DMSO-*d*_6_) δ = 11.85 (br s, 1H), 10.66 (br s, 1H), 9.77 (br s, 1H), 8.07 (d, *J* = 8.8 Hz, 2H), 7.85 (d, *J* = 8.3 Hz, 2H), 7.68 (d, *J* = 8.3 Hz, 2H), 7.58 (d, *J* = 8.8 Hz, 2H), 7.32 (d, *J* = 7.8 Hz, 2H), 6.83 (d, *J* = 8.8 Hz, 2H), 6.52 (br s, 2H), 2.36 (s, 3H) ppm; ^13^C-NMR (101 MHz, DMSO-*d*_6_) δ = 20.7, 115.5, 116.0, 122.5, 126.5, 126.8, 128.3, 129.7, 132.3, 136.1, 137.7, 143.3, 145.9, 158.0, 165.0 ppm; HRMS (HESI, *m*/*z*) calcd. for C_23_H_20_N_6_O_2_ (412.45) [M − H]^−^ 411.1570, found 411.1568.

*N-(5-Amino-4-((4-hydroxyphenyl)diazenyl)-1H-pyrazol-3-yl)-[1,1′-biphenyl]-4-carboxamide* (**12e**): Yield 82 mg (82%) as a bright orange solid; m.p. 249–253 °C; ^1^H-NMR (400 MHz, DMSO-*d*_6_) δ = 11.86 (br s, 1H), 10.67 (br s, 1H), 9.77 (s, 1H), 8.09 (d, *J* = 7.8 Hz, 2H), 7.88 (d, *J* = 8.2 Hz, 2H), 7.78 (d, *J* = 8.2 Hz, 2H), 7.59 (d, *J* = 8.7 Hz, 2H), 7.52 (t, *J* = 7.6 Hz, 2H), 7.43 (t, *J* = 7.3 Hz, 1H), 6.83 (d, *J* = 8.7 Hz, 2H), 6.54 (br s, 2H) ppm; ^13^C-NMR (101 MHz, DMSO-*d*_6_) δ = 115.6, 116.0, 122.5, 126.9, 127.0, 128.3, 128.4, 129.1, 132.6, 139.1, 143.4, 145.9, 158.1, 165.0 ppm; HRMS (HESI, *m*/*z*) calcd. for C_22_H_18_N_6_O_2_ (398.43) [M − H]^−^ 397.1413, found 397.1406.

*N-(5-Amino-4-((4-hydroxyphenyl)diazenyl)-1H-pyrazol-3-yl)-4′-methoxy-[1,1′-biphenyl]-4-carboxamide* (**12f**): Yield 92 mg (86%) as an orange-red solid; m.p. 220–224 °C; ^1^H-NMR (400 MHz, DMSO-*d*_6_) δ = 1.85 (br s, 1H), 10.65 (br s, 1H), 9.77 (br s., 1H), 8.05 (d, *J* = 8.3 Hz, 2H), 7.83 (d, *J* = 8.3 Hz, 2H), 7.74 (d, *J* = 9.3 Hz, 2H), 7.59 (d, *J* = 8.8 Hz, 2H), 7.07 (d, *J* = 8.8 Hz, 2H), 6.83 (d, *J* = 8.8 Hz, 2H), 6.53 (br s, 2H), 3.82 (s, 3H) ppm; ^13^C-NMR (101 MHz, DMSO-*d*_6_) δ = 55.2, 114.5, 115.5, 116.0, 122.5, 126.2, 128.1, 128.3, 131.3, 131.8, 143.1, 145.9, 158.0, 159.5, 165.0 ppm; HRMS (HESI, *m*/*z*) calcd. for C_23_H_20_N_6_O_3_ (428.45) [M − H]^−^ 427.1519, found 427.1513.

*N-(5-Amino-4-((4-hydroxyphenyl)diazenyl)-1H-pyrazol-3-yl)-2′-methoxy-[1,1′-biphenyl]-4-carboxamide* (**12g**): Yield 100 mg (93%) as an orange solid; m.p. 188–192 °C; ^1^H-NMR (400 MHz, DMSO-*d*_6_) δ = 11.90 (br s, 1H), 10.66 (br s, 1H), 9.79 (s, 1H), 8.02 (d, *J* = 8.2 Hz, 2H), 7.67 (d, *J* = 8.2 Hz, 2H), 7.58 (d, *J* = 8.7 Hz, 2H), 7.43–7.35 (m, 2H), 7.15 (dd, *J* = 0.9, 7.8 Hz, 1H), 7.07 (dt, *J* = 0.9, 7.3 Hz, 1H), 6.83 (d, *J* = 8.7 Hz, 2H), 6.52 (br s, 2H), 3.80 (s, 3H) ppm; ^13^C-NMR (101 MHz, DMSO-*d*_6_) δ = 55.6, 111.9, 115.6, 116.0, 121.0, 122.5, 127.4, 128.8, 129.5, 129.6, 130.5, 132.1, 141.8, 144.1, 145.9, 156.2, 158.1, 165.2 ppm; HRMS (HESI, *m*/*z*) calcd. for C_23_H_20_N_6_O_3_ (428.45) [M − H]^−^ 427.1519, found 427.1515 [M − H]^−^.

*N-(5-Amino-4-((4-hydroxyphenyl)diazenyl)-1H-pyrazol-3-yl)-4′-nitro-[1,1′-biphenyl]-4-carboxamide* (**12h**): After 12 h the same amount of 4-nitroboronic acid and XPhos Pd G2 was added and the reaction was proceeded for next 24 h. Yield 50 mg (45%) as an orange solid; m.p. 187–192 °C; ^1^H-NMR (400 MHz, DMSO-*d*_6_) δ = 11.80 (br s, 1H), 10.66 (br s, 1H), 9.74 (br s, 1H), 8.35 (d, *J* = 8.7 Hz, 2H), 8.13 (d, *J* = 8.7 Hz, 2H), 8.08 (d, *J* = 9.2 Hz, 2H), 8.00 (d, *J* = 8.2 Hz, 2H), 7.57 (d, *J* = 8.2 Hz, 2H), 6.81 (d, *J* = 9.2 Hz, 2H), 6.61 (br s, 2H) ppm; ^13^C-NMR (101 MHz, DMSO-*d*_6_) δ = 115.5, 122.5, 124.2, 127.5, 128.2, 128.5 134.0, 140.9, 145.5, 145.9, 147.1, 158.0, 164.8 ppm; HRMS (HESI, *m*/*z*) calcd. for C_22_H_17_N_7_O_4_ (443.42) [M + H]^+^ 444.1420, found 444.1416.

*N-(5-Amino-4-((4-hydroxyphenyl)diazenyl)-1H-pyrazol-3-yl)-4-(furan-3-yl)benzamide* (**12i**): Yield 85 mg (86%) as an orange solid; m.p. 266–271 °C; ^1^H-NMR (400 MHz, DMSO-*d*_6_) δ = 11.86 (br s, 1H), 10.61 (br s, 1H), 9.79 (s, 1H), 8.36 (s, 1H), 8.01 (d, *J* = 8.2 Hz, 2H), 7.82 (d, *J* = 8.2 Hz, 2H), 7.80 (t, *J* = 1.6 Hz, 1H), 7.57 (d, *J* = 8.7 Hz, 2H), 7.10–7.07 (m, 1H), 6.83 (d, *J* = 8.7 Hz, 2H), 6.53 (br s, 2H) ppm; ^13^C-NMR (101 MHz, DMSO-*d*_6_) δ = 108.7, 115.6, 116.0, 122.5, 125.2, 125.6, 128.3, 132.0, 135.5, 140.6, 143.9, 144.7, 145.9, 158.1, 165.0 ppm; HRMS (HESI, *m*/*z*) calcd. for C_20_H_16_N_6_O_3_ (388.39) [M + H]^+^ 389.1362, found 389.1356.

*N-(5-Amino-4-((4-hydroxyphenyl)diazenyl)-1H-pyrazol-3-yl)-4-(thiophen-3-yl)benzamide (***12j***)*: Yield 97 mg (96%) as a yellow-orange solid; m.p. 272–274 °C; ^1^H-NMR (400 MHz, DMSO-*d*_6_) δ = 11.85 (br s, 1H), 10.63 (br s, 1H), 9.77 (s, 1H), 8.08 (dd, *J* = 1.6, 2.5 Hz, 1H), 8.03 (d, *J* = 8.2 Hz, 2H), 7.93 (d, *J* = 8.2 Hz, 2H), 7.72–7.67 (m, 2H), 7.57 (d, *J* = 8.7 Hz, 2H), 6.82 (d, *J* = 9.2 Hz, 2H), 6.54 (br s, 2H) ppm; ^13^C-NMR (101 MHz, DMSO-*d*_6_) δ = 115.5, 116.0, 122.4, 122.7, 126.1, 126.3, 127.5, 128.4, 132.1, 138.3, 140.4, 145.9, 158.0, 164.9 ppm; HRMS (HESI, *m*/*z*) calcd. for C_20_H_16_N_6_O_2_S (404.45) [M − H]^−^ 403.0977, found 403.0975.

*N-(5-Amino-4-((4-hydroxyphenyl)diazenyl)-1H-pyrazol-3-yl)-4′-methoxy-[1,1′-biphenyl]-3-carboxamide* (**12k**): Yield 43 mg (40%) as a dark orange solid; m.p. 192–196 °C; ^1^H-NMR (400 MHz, DMSO-*d*_6_) δ = 11.99 (br s, 1H), 10.70 (br s, 1H), 9.78 (br s, 1H), 8.21 (s, 1H), 7.90 (d, *J* = 7.8 Hz, 1H), 7.86 (d, *J* = 7.8 Hz, 1H), 7.73 (d, *J* = 8.7 Hz, 2H), 7.61 (t, *J* = 7.8 Hz, 1H), 7.53 (d, *J* = 8.7 Hz, 2H), 7.07 (d, *J* = 8.7 Hz, 2H), 6.78 (d, *J* = 8.7 Hz, 2H), 6.56 (br s, 2H), 3.82 (s, 3H) ppm; ^13^C-NMR (101 MHz, DMSO-*d*_6_) δ = 55.3, 114.5, 115.5, 116.1, 122.5, 125.3, 126.1, 127.9, 128.1, 129.3, 129.6, 131.8, 134.5, 140.2, 140.8, 143.7, 145.9, 158.0, 159.3, 165.4 ppm; HRMS (HESI, *m*/*z*) calcd. for C_23_H_20_N_6_O_3_ (428.45) [M + H]^+^ 429.1675, found 429.1674.

*N-(5-Amino-4-((4-hydroxyphenyl)diazenyl)-1H-pyrazol-3-yl)-2′-methoxy-[1,1′-biphenyl]-3-carboxamide* (**12l**): Yield 70 mg (65%) as a brown solid; m.p. 175–180 °C; ^1^H-NMR (400 MHz, DMSO-*d*_6_) δ = 11.84 (br s, 1H), 10.74 (br s, 1H), 9.77 (br s, 1H), 8.06 (s, 1H), 7.93 (d, *J* = 7.8 Hz, 1H), 7.72 (d, *J* = 7.8 Hz, 1H), 7.60 (t, *J* = 7.6 Hz, 1H), 7.51 (d, *J* = 8.7 Hz, 2H), 7.41 (d, *J* = 7.3 Hz, 2H), 7.17 (d, *J* = 8.7 Hz, 1H), 7.09 (t, *J* = 7.6 Hz, 1H), 6.75 (d, *J* = 8.7 Hz, 2H), 6.50 (br s, 2H), 3.77 (s, 3H) ppm; ^13^C-NMR (101 MHz, DMSO-*d*_6_) δ = 55.6, 111.8, 115.5, 120.9, 122.4, 126.3, 128.2, 128.5, 128.9, 129.5, 130.6, 132.9, 133.7, 138.5, 144.5, 145.8, 156.1, 158.0, 165.1 ppm; HRMS (HESI, *m*/*z*) calcd. for C_23_H_20_N_6_O_3_ (428.45) [M + H]^+^ 429.1675, found 429.1672.

*N-(5-Amino-4-((4-hydroxyphenyl)diazenyl)-1H-pyrazol-3-yl)-3-(furan-3-yl)benzamide* (**12m**): Yield 49 mg (50%) as an orange solid; m.p. 204–208 °C; ^1^H-NMR (400 MHz, DMSO-*d*_6_) δ = 11.81 (br s, 1H), 10.58 (br s, 1H), 9.76 (br s, 1H), 8.29 (t, *J* = 1.0 Hz, 1H), 8.21 (t, *J* = 1.6 Hz, 1H), 7.86 (dd, *J* = 1.6, 7.6 Hz, 2H), 7.80 (t, *J* = 1.6 Hz, 1H), 7.57 (t, *J* = 7.8 Hz, 1H), 7.53 (d, *J* = 8.7 Hz, 2H), 7.08–7.04 (m, 1H), 6.79 (d, *J* = 8.7 Hz, 2H), 6.56 (br s, 2H) ppm; ^13^C-NMR (101 MHz, DMSO-*d*_6_) δ = 108.8, 115.5, 116.2, 122.5, 124.6, 125.3, 126.2, 128.9, 129.2, 132.4, 134.5, 139.9, 144.6, 145.9, 158.0, 165.4 ppm; HRMS (HESI, *m*/*z*) calcd. for C_20_H_16_N_6_O_3_ (388.39) [M + H]^+^ 389.1362, found 389.1357.

*N-(5-Amino-4-((4-hydroxyphenyl)diazenyl)-1H-pyrazol-3-yl)-3-(thiophen-3-yl)benzamide* (**12n**): Yield 61 mg (60%) as a bright orange solid; m.p. 278–282 °C; ^1^H-NMR (400 MHz, DMSO-*d*_6_) δ = 11.94 (br s, 1H), 10.66 (br s, 1H), 9.78 (br s, 1H), 8.32 (s, 1H), 8.02–8.00 (m, 1H), 7.96 (d, *J* = 7.8 Hz, 1H), 7.89 (d, *J* = 7.3 Hz, 1H), 7.72–7.69 (m, 1H), 7.67 (d, *J* = 5.0 Hz, 1H), 7.60 (t, *J* = 7.8 Hz, 1H), 7.54 (d, *J* = 8.2 Hz, 2H), 6.78 (d, *J* = 8.7 Hz, 2H), 6.58 (br s, 2H) ppm; ^13^C-NMR (101 MHz, DMSO-*d*_6_) δ = 115.5, 116.2, 121.9, 122.5, 125.1, 126.3, 126.5, 127.5, 129.3, 129.5, 134.6, 135.5, 140.8, 143.5, 145.9, 158.0, 165.4 ppm; HRMS (HESI, *m*/*z*) calcd. for C_20_H_16_N_6_O_2_S (404.45) [M − H]^−^ 403.0977, found 403.0971. 

## 4. Conclusions

To conclude, we developed the simple Boc-protection method of pyrazole **1** (CAN508) to access amino-benzoylated pyrazoles **11** and **12**. The positions of the Boc groups in pyrazoles **3** and **5** were unequivocally determined by single crystal X-ray analysis. Further modification of bromopyrazoles **11c** and **12b** via Suzuki-Miyaura coupling showed the relative robustness of the catalyst and proved the possibility to carry out the coupling in the last step even with the unprotected amino-benzoylated pyrazole system. The coupling reaction gave better yields with *para*-substituted bromobenzoylpyrazole **11c**. Moreover, the use of Boc protecting groups enabled for directly synthesizing pyrazoles **12d**–**n** without an additional deprotection step.

## Figures and Tables

**Table 1:**
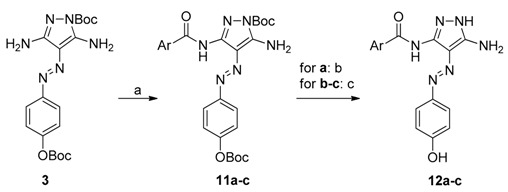
Benzoylation of pyrazole **3** and subsequent deprotection ^1^.

Entry	Pyrazole	Ar	Benzoylation [%] ^2^	Deprotection [%] ^2^
1	**11a/12a**	Phenyl	-	92
2	**11b/12b**	3-Bromophenyl	-	80
3	**11c/12c**	4-Bromophenyl	84	95

^1^ Reagents and conditions: (a) ArCOCl, pyridine, r.t., 3 h; (b) 10% TFA in DCM, r.t., 2.5 h; (c) K_3_PO_4_, 1,4-dioxane/H_2_O (4:1), 100 °C, 1.5 h. ^2^ Isolated yield.

**Table 2:**
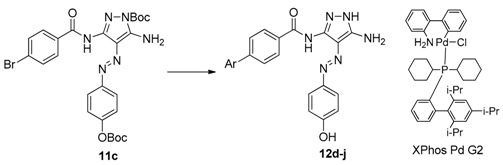
Arylation of pyrazole **11c** by Suzuki-Miyaura coupling ^1^.

Entry	Pyrazole	Ar	Yield [%] ^2^
1	**12d**	Tolyl	88
2	**12e**	Phenyl	82
3	**12f**	4-MeO-phenyl	86
4	**12g**	2-MeO-phenyl	93
5	**12h**	4-NO_2_-phenyl	45 ^3^
6	**12i**	Furan-3-yl	86
7	**12j**	Thiophen-3-yl	96

^1^ Reagents and conditions: XPhos Pd G2 (5 mol %), K_3_PO_4_ (4 equiv.), (hetero)arylboronic acid (2 equiv.), 1,4-dioxane/water 4:1, 100 °C, 24 h. ^2^ Isolated yield. ^3^ After 12 h, the same amount of 4-nitroboronic acid and XPhos Pd G2 was added and the reaction was proceeded for the next 24 h.

**Table 3:**
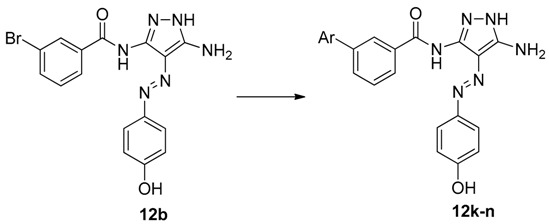
Arylation of pyrazole **12b** by Suzuki-Miyaura coupling ^1^.

Entry	Pyrazole	Ar	Yield [%] ^2^
1	**12k**	4-MeO-phenyl	41
2	**12l**	2-MeO-phenyl	65
3	**12m**	Furan-3-yl	50
4	**12n**	Thiophen-3-yl	60

^1^ Reagents and conditions: XPhos Pd G2 (5 mol %), K_3_PO_4_ (4 equiv.), (hetero)arylboronic acid (2 equiv.), 1,4-dioxane/water 4:1, 100 °C, 24 h. ^2^ Isolated yield.

## Supplementary Materials

The following are available online, the copies of ^1^H and ^13^C-NMR spectra, crystallographic data for structures **3** and **5**. CCDC 1453321 and CCDC 1453320 contain the supplementary crystallographic data for this paper. These data can be obtained free of charge via http://www.ccdc.cam.ac.uk/conts/retrieving.html (or from the CCDC, 12 Union Road, Cambridge CB2 1EZ, UK; Fax: +44 1223 336033; E-mail: deposit@ccdc.cam.ac.uk).
