# Supplementary material for: Modification of Boc-Protected CAN508 via Acylation and Suzuki-Miyaura Coupling
Source: Molecules. 2018 Jan 12;23(1):149. doi: 10.3390/molecules23010149 (PMC6017724; doi:10.3390/molecules23010149)
Supplement: Supplementary file 1 [file molecules-23-00149-s001.pdf]

Supplementary File

Modification of Boc-protected CAN508 via acylation and Suzuki-Miyaura Coupling

Martin Pisár [1], Eva Schütznerová [2], Filip Hančík [1], Igor Popa [3], Zdeněk Trávníček [3] and Petr Cankař [1,*]

[1] Department of Organic Chemistry, Faculty of Science, Palacký University, 17. listopadu 1192/12, 771 46 Olomouc, Czech Republic; martin.pisar01@upol.cz (M.P.); Filda.Hancik@seznam.cz (F.H.)

[2] Institute of Molecular and Translation Medicine, Faculty of Medicine, Palacký University, Hněvotínská 5, 77900 Olomouc, Czech Republic; eva.schutznerova@upol.cz

[3] Department of Inorganic Chemistry, Faculty of Science, Palacký University, 17. listopadu 1192/12, 771 46 Olomouc, Czech Republic; igor.popa@upol.cz (I.P.); zdenek.travnicek@upol.cz (Z.T.)

* Correspondence: petr.cankar@upol.cz; Tel.: +42-058-563-4437

Table of Contents

1. ^{1}H and ^{13}C NMR spectra ……………………………………………………..………. S2

2. Crystallographic data for structures **5** and **3** ………………………………….......... S23

^{1}H and ^{13}C NMR spectra of 3

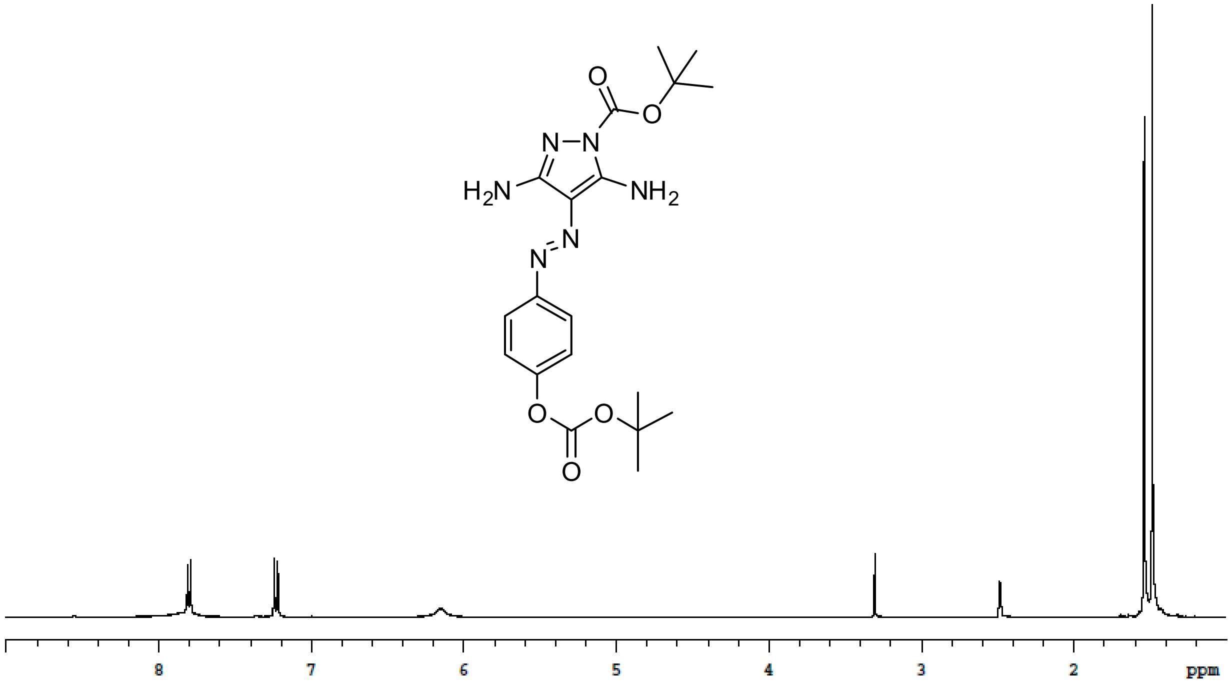

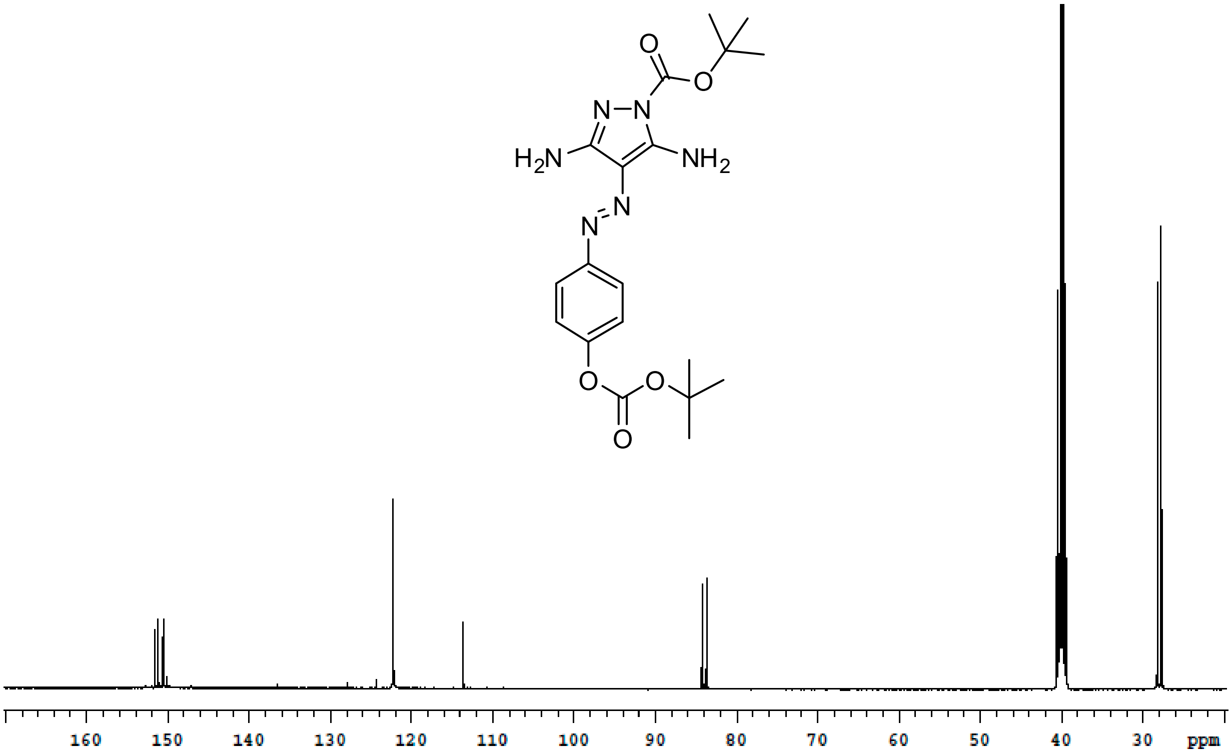

^{1}H and ^{13}C NMR spectra of 5

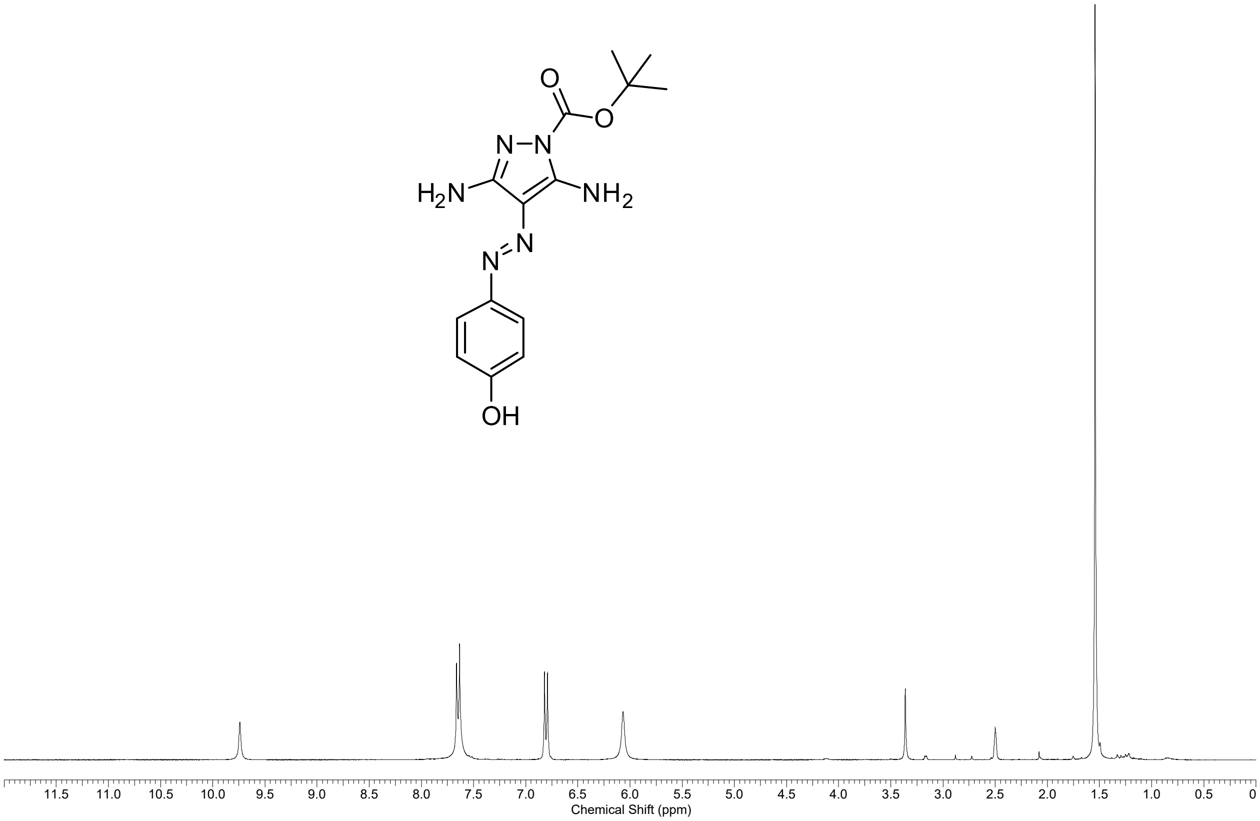

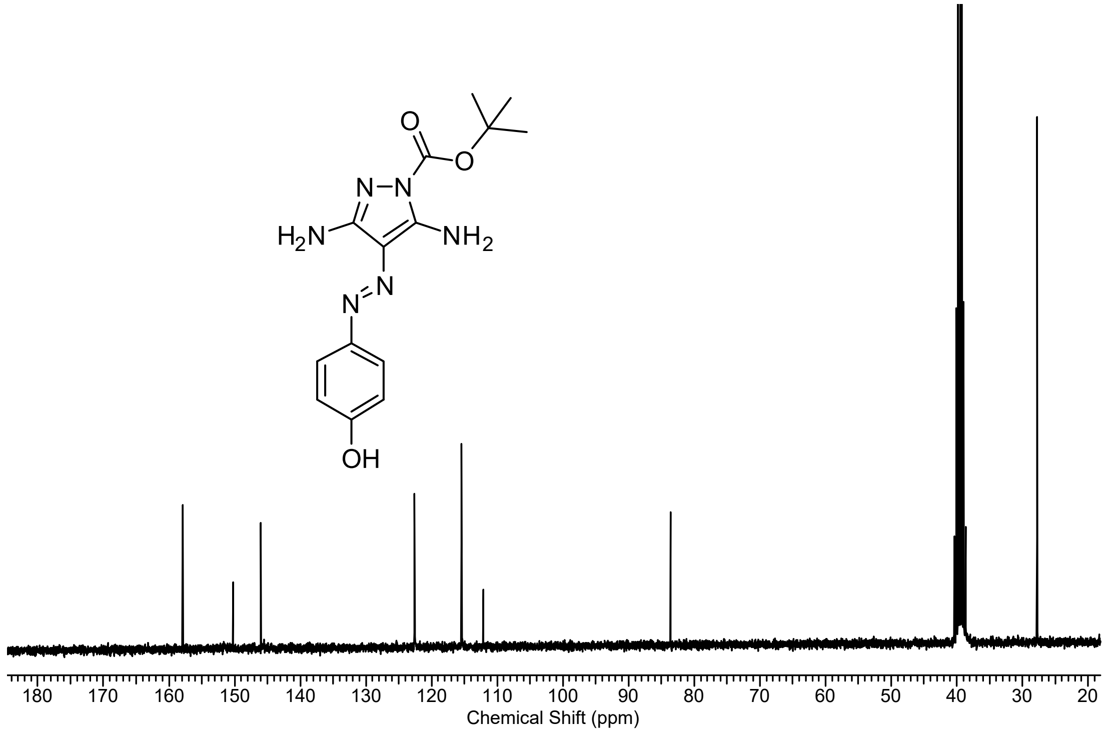

^{1}H and ^{13}C NMR spectra of 6

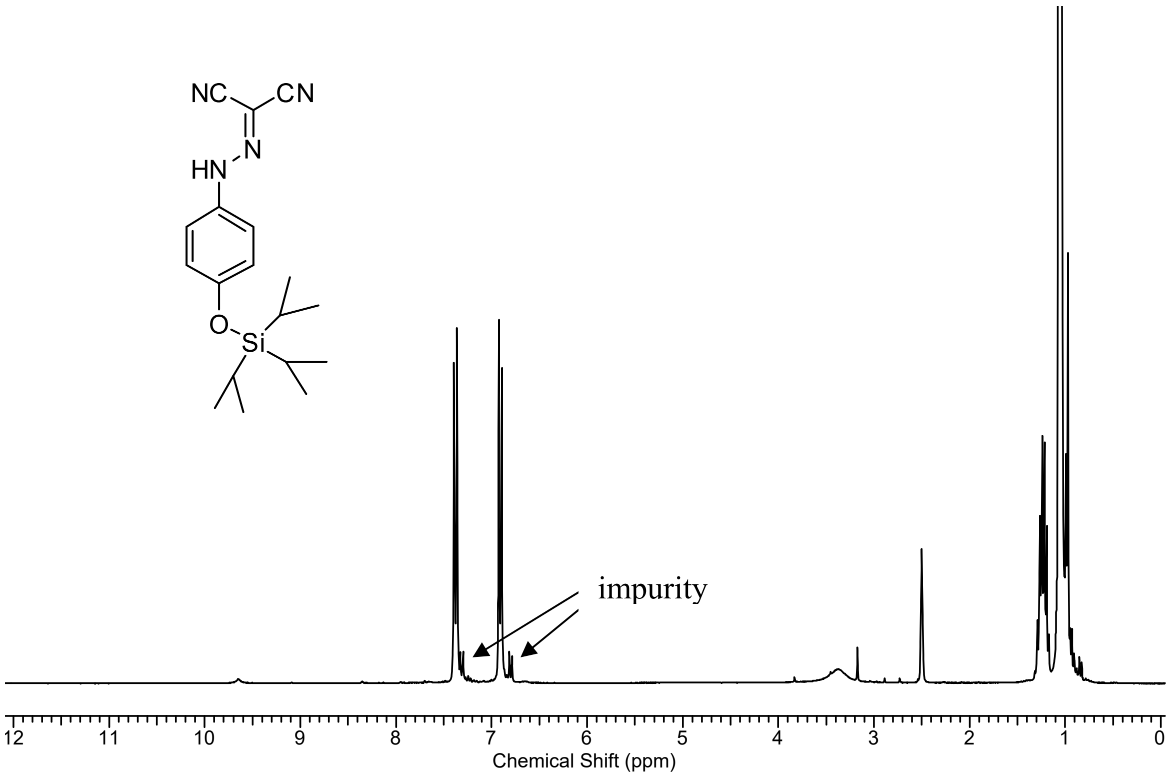

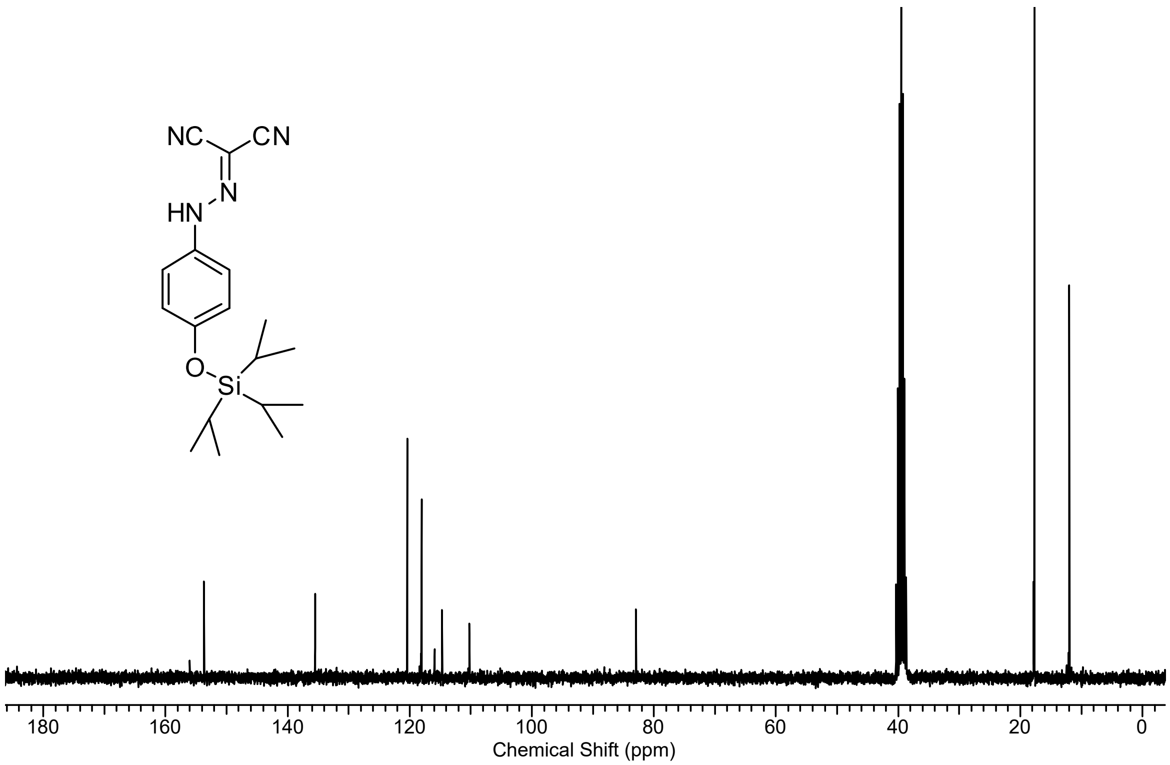

^{1}H and ^{13}C NMR spectra of 7

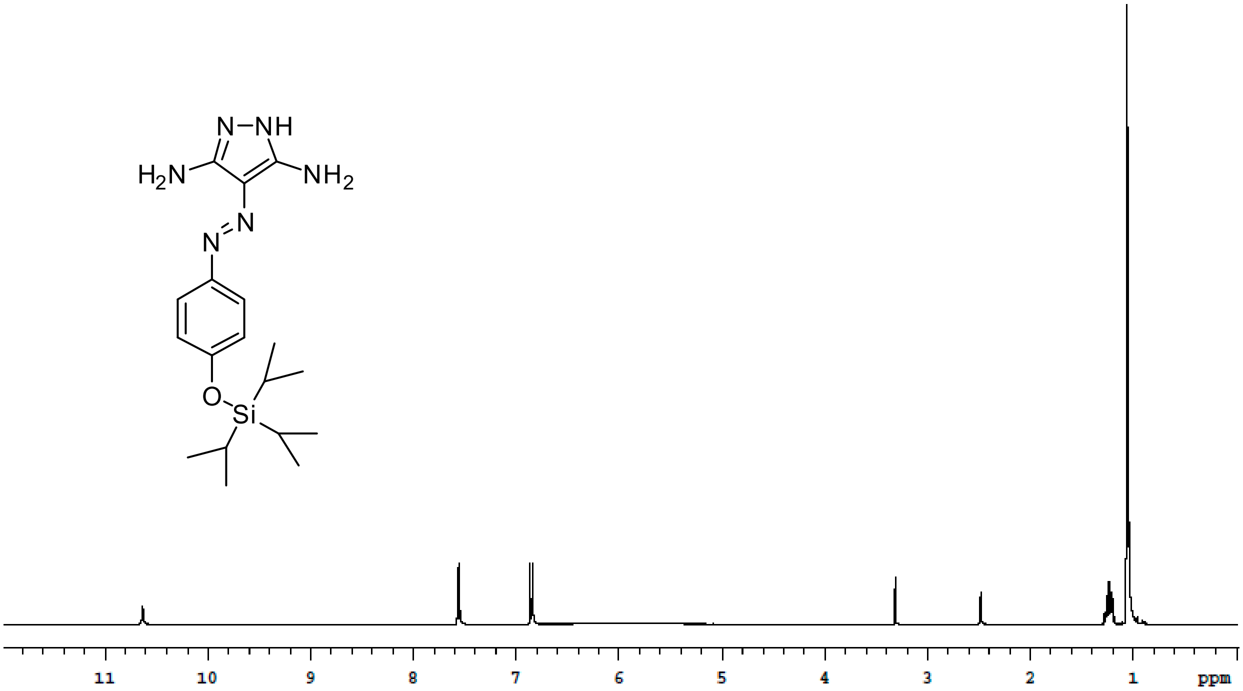

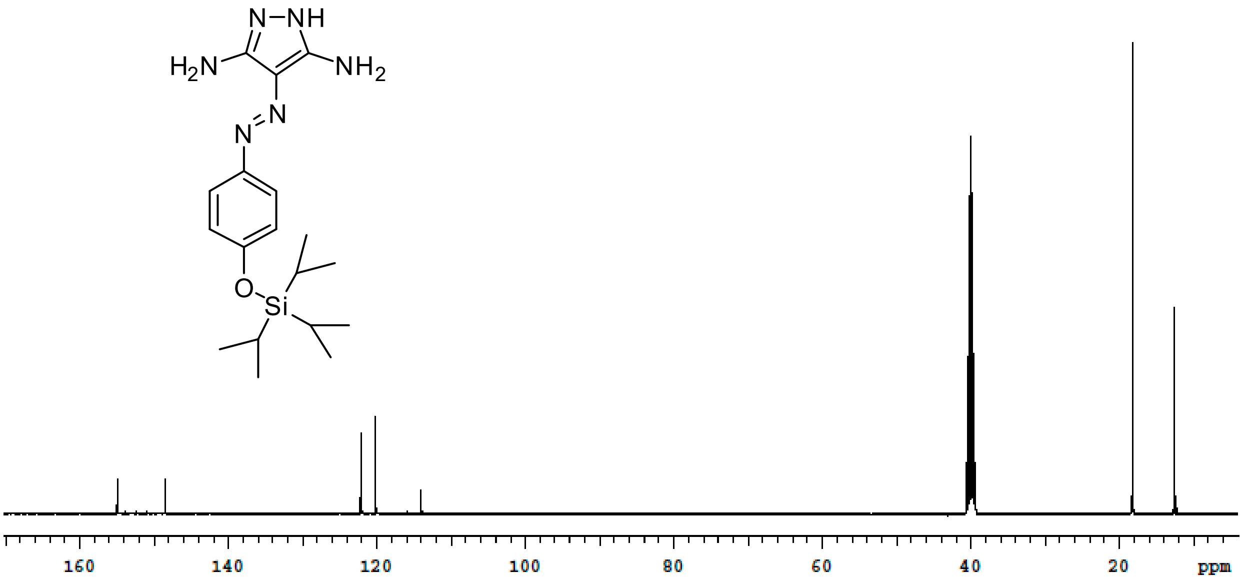

^{1}H and ^{13}C NMR spectra of 8

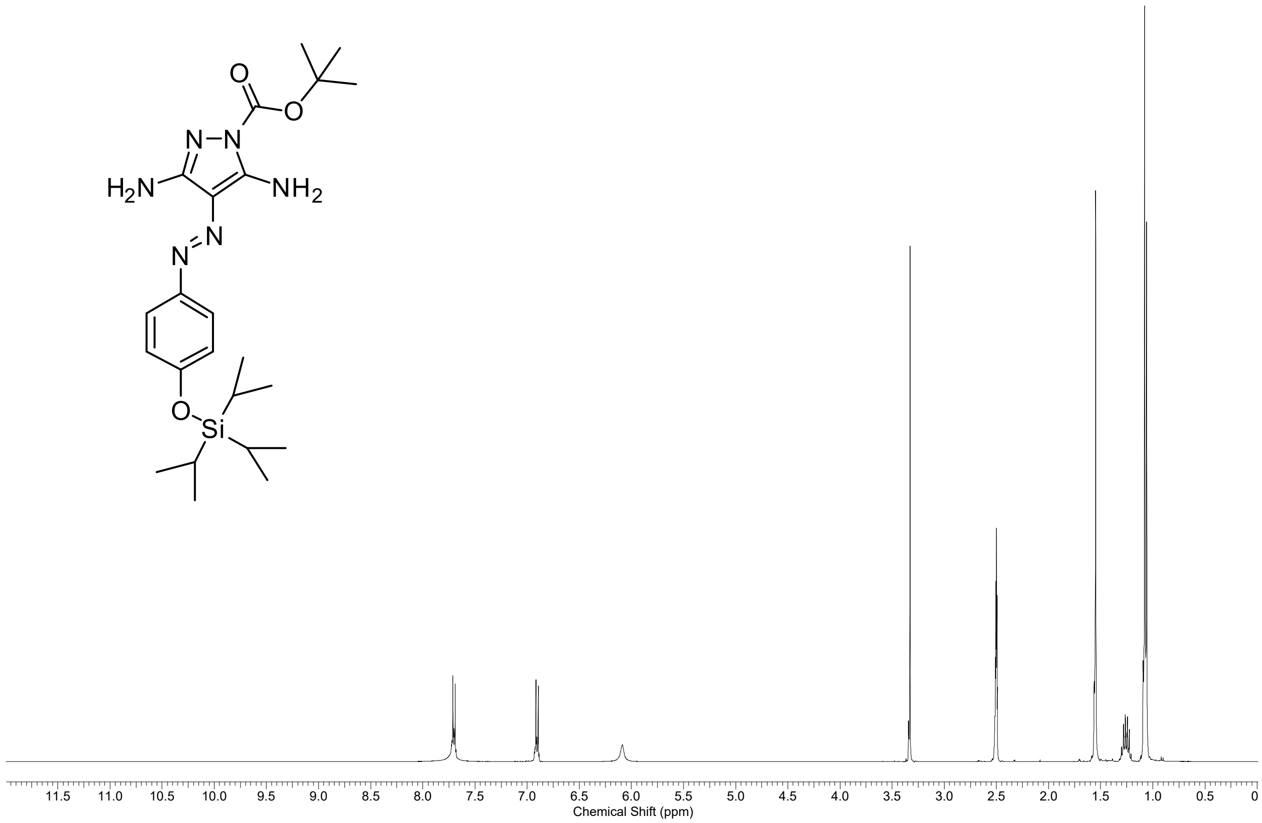

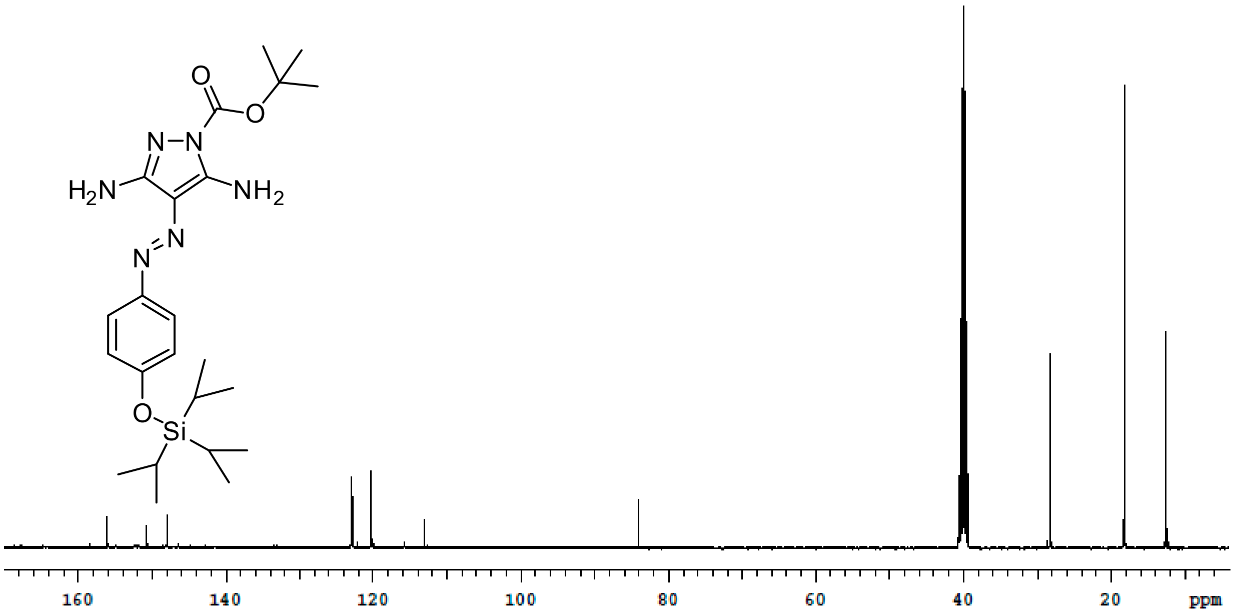

^{1}H and ^{13}C NMR spectra of 9

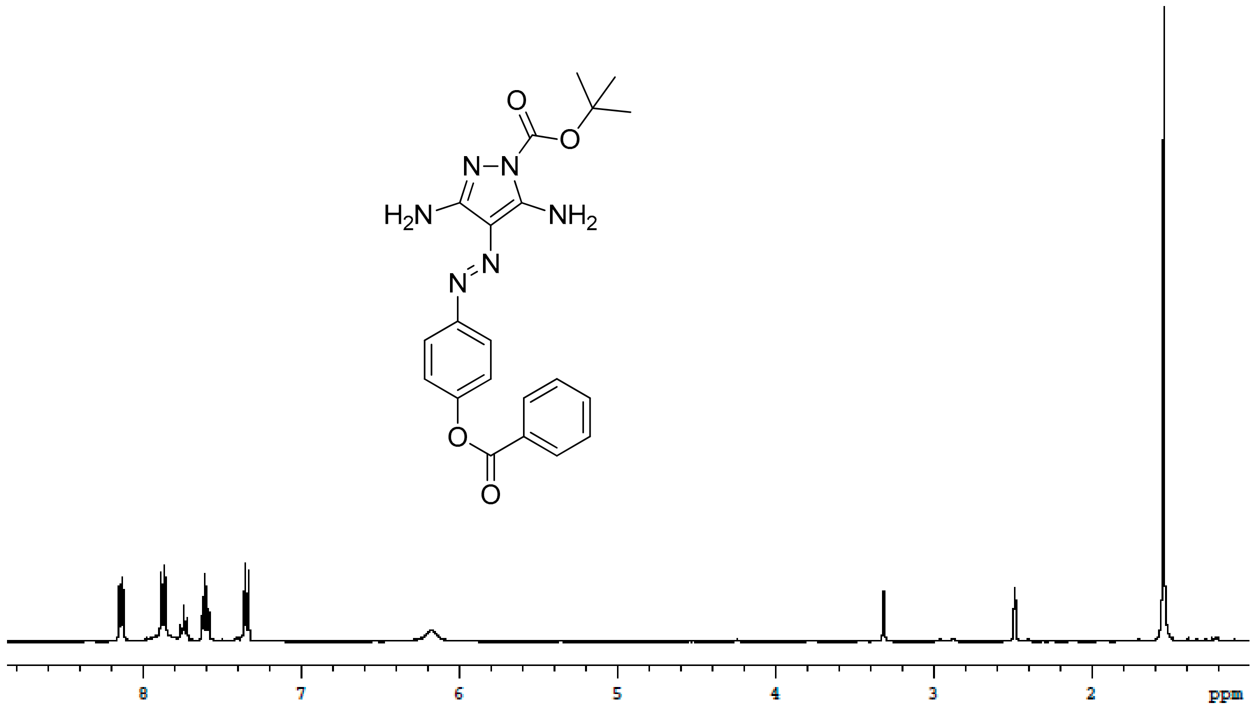

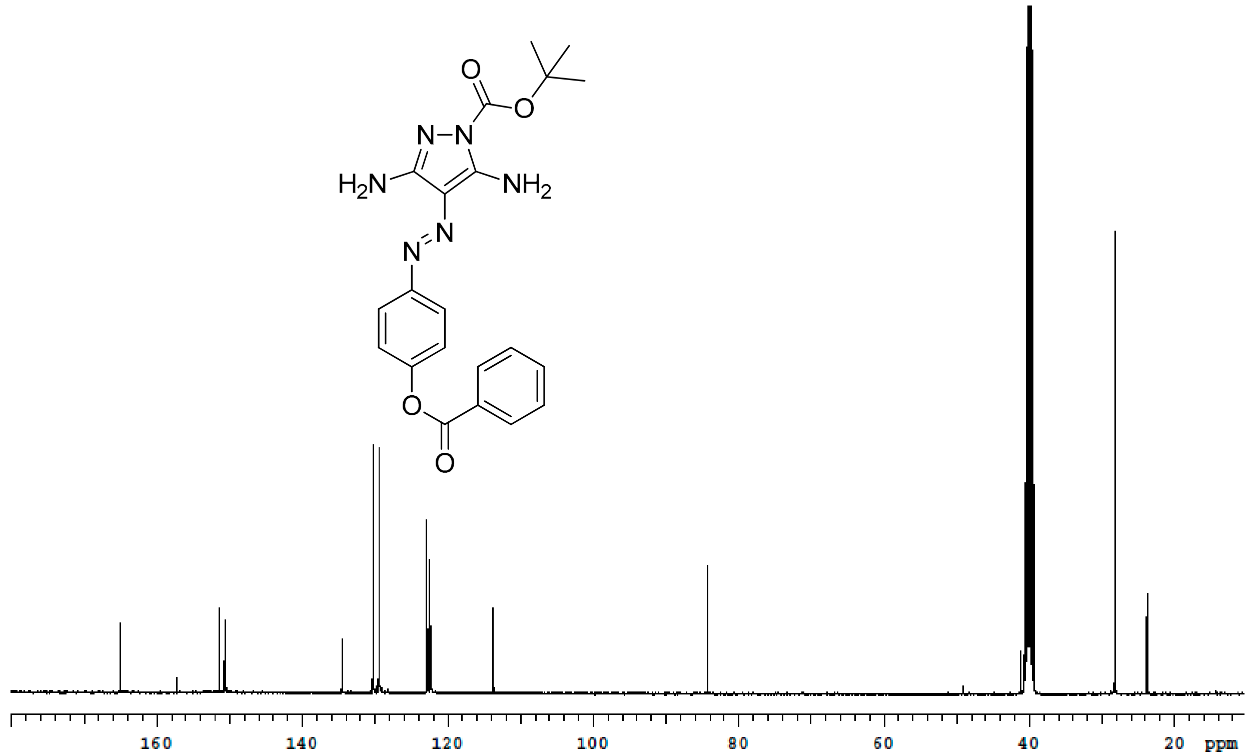

^{1}H and ^{13}C NMR spectra of 10

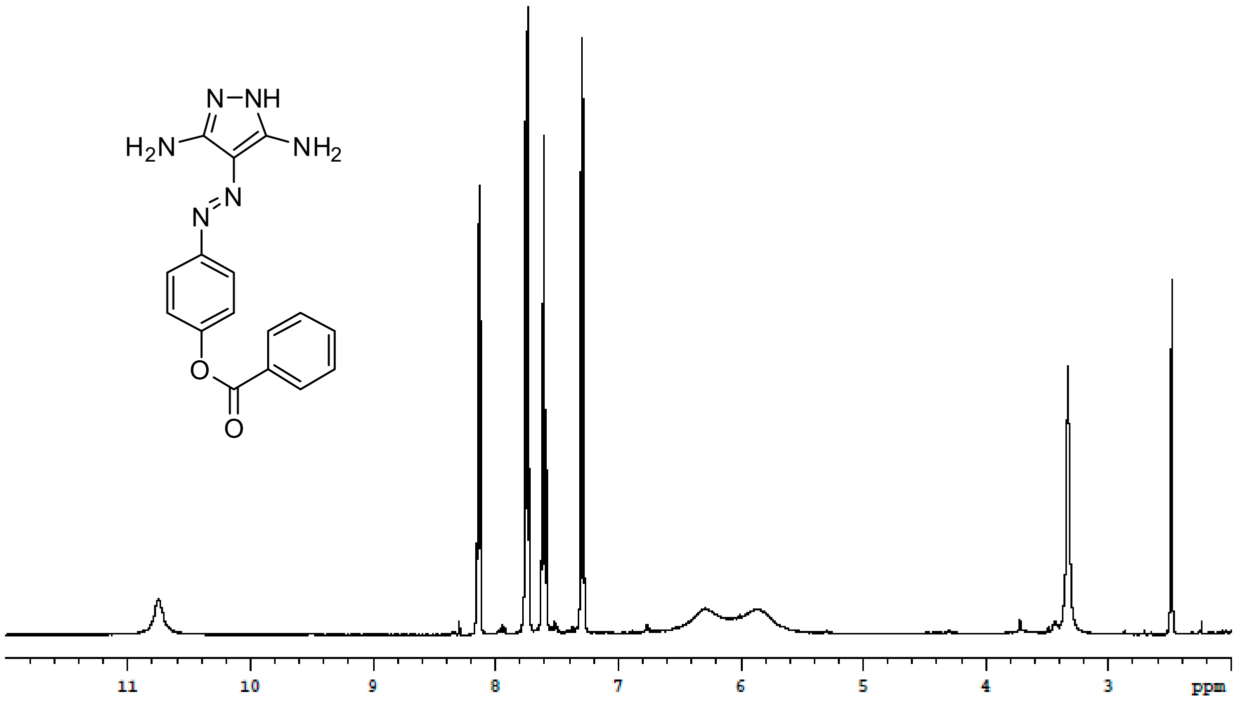

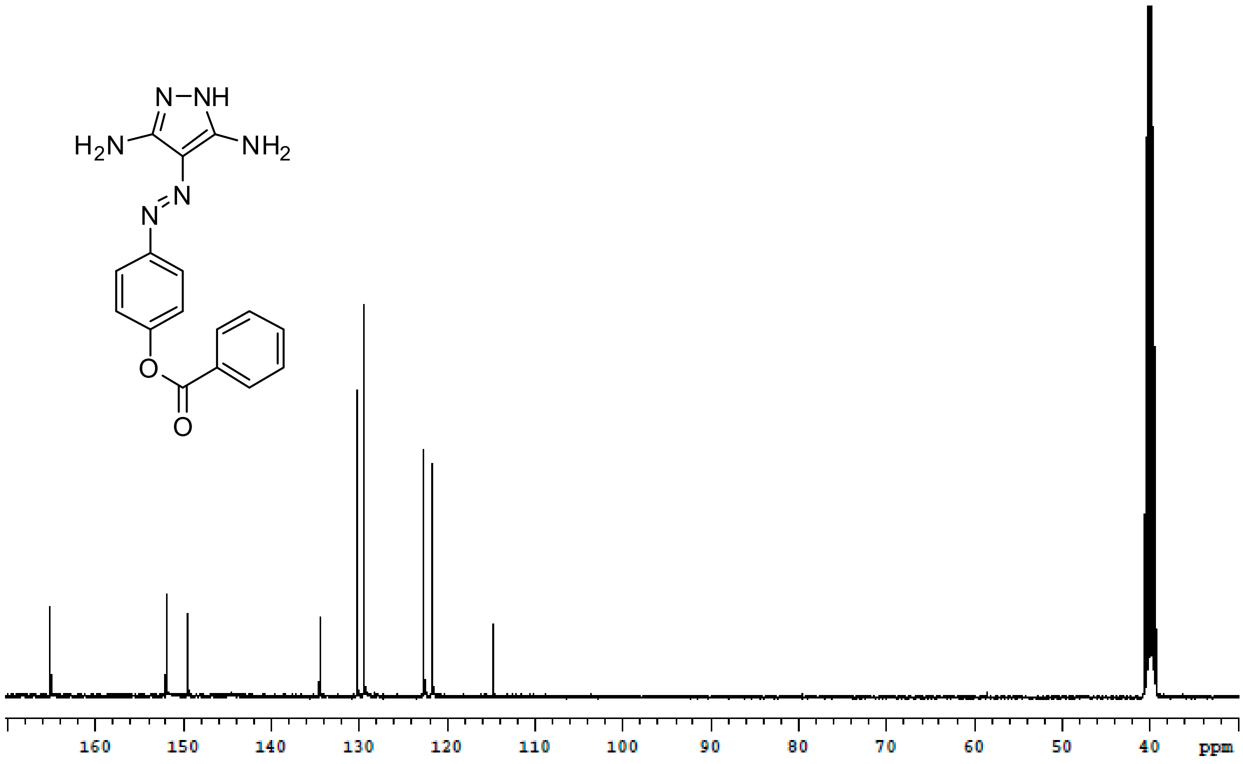

^{1}H and ^{13}C NMR spectra of 11c

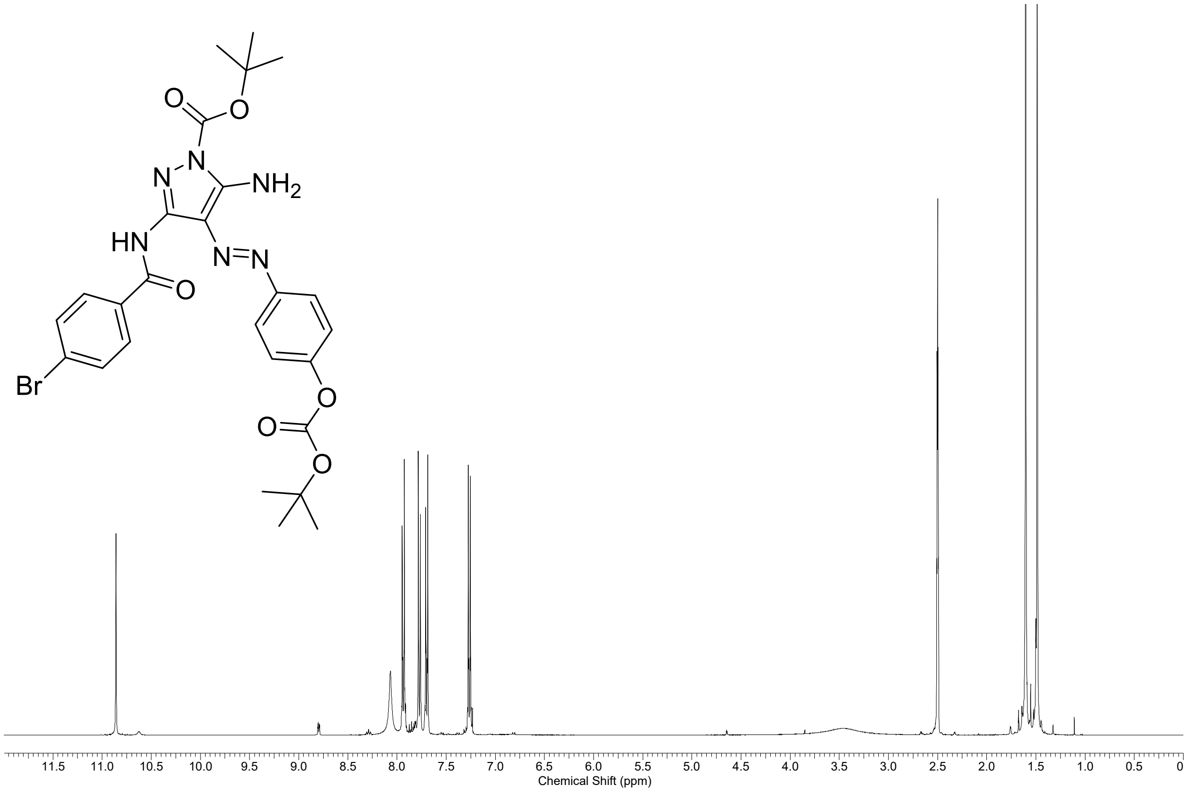

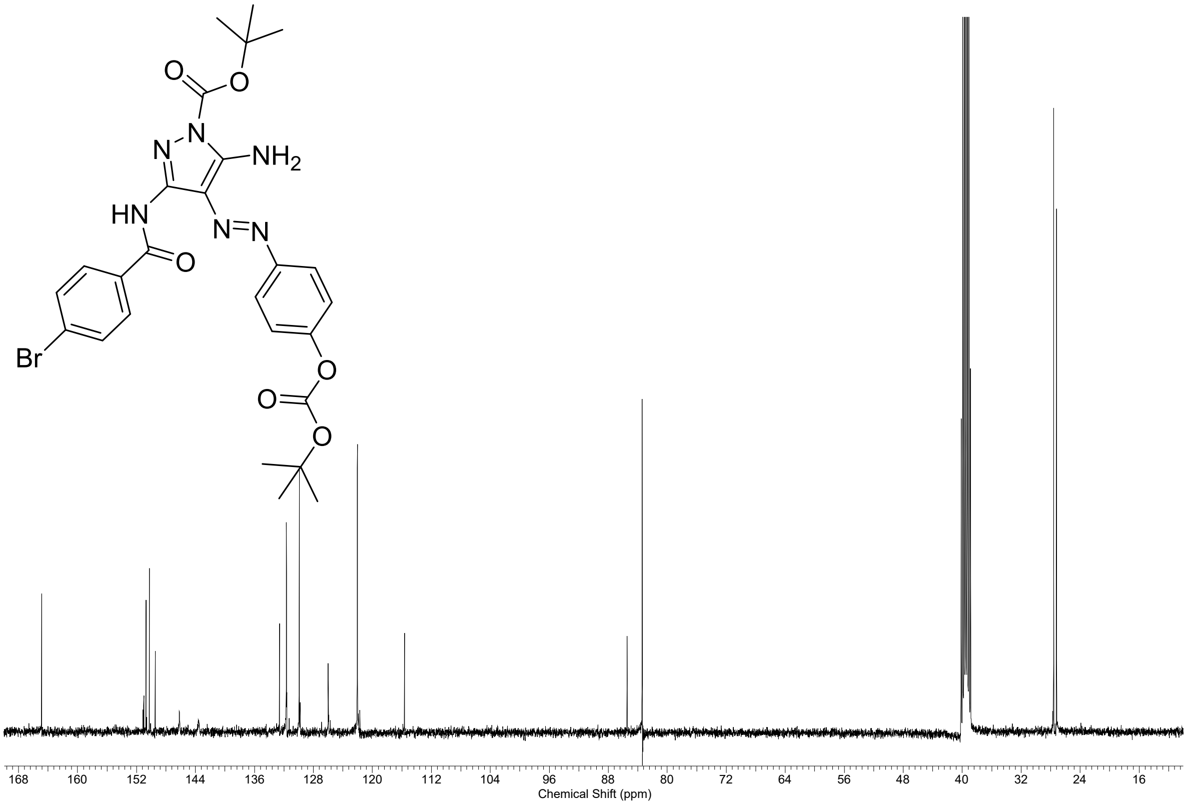

^{1}H and ^{13}C NMR spectra of 12b

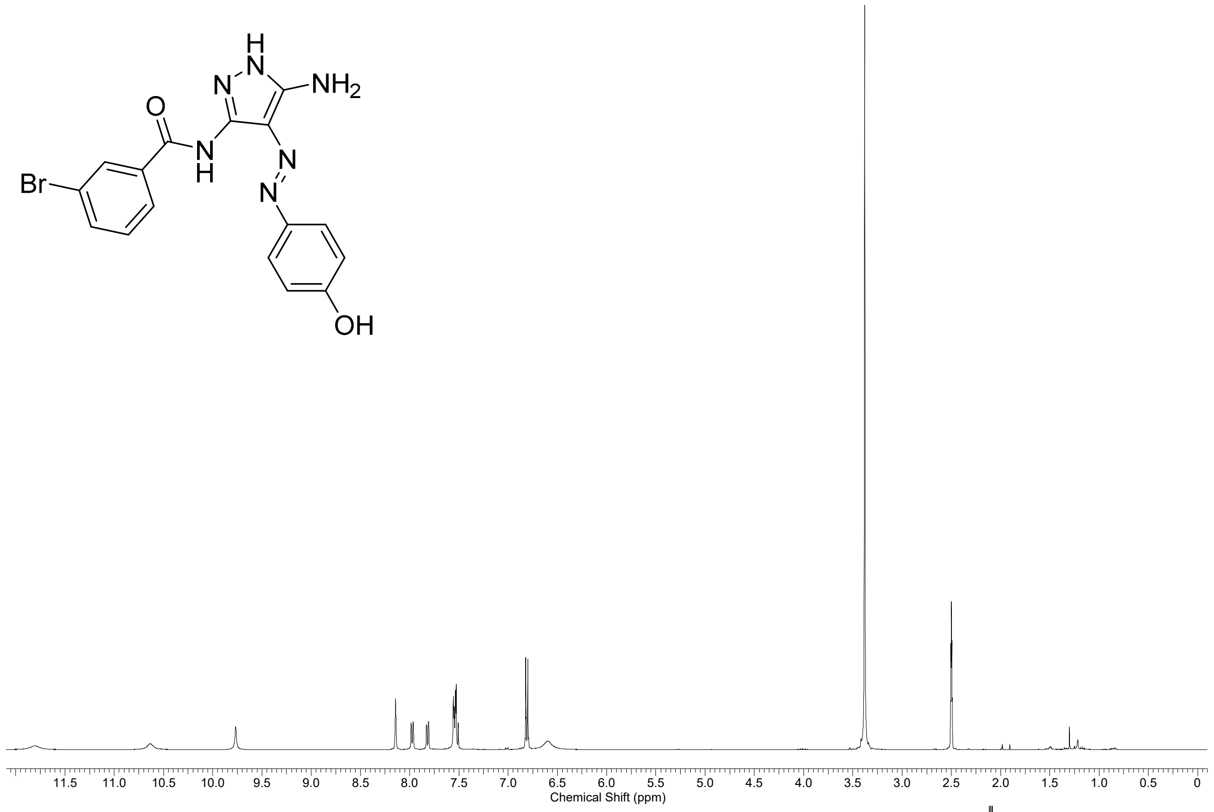

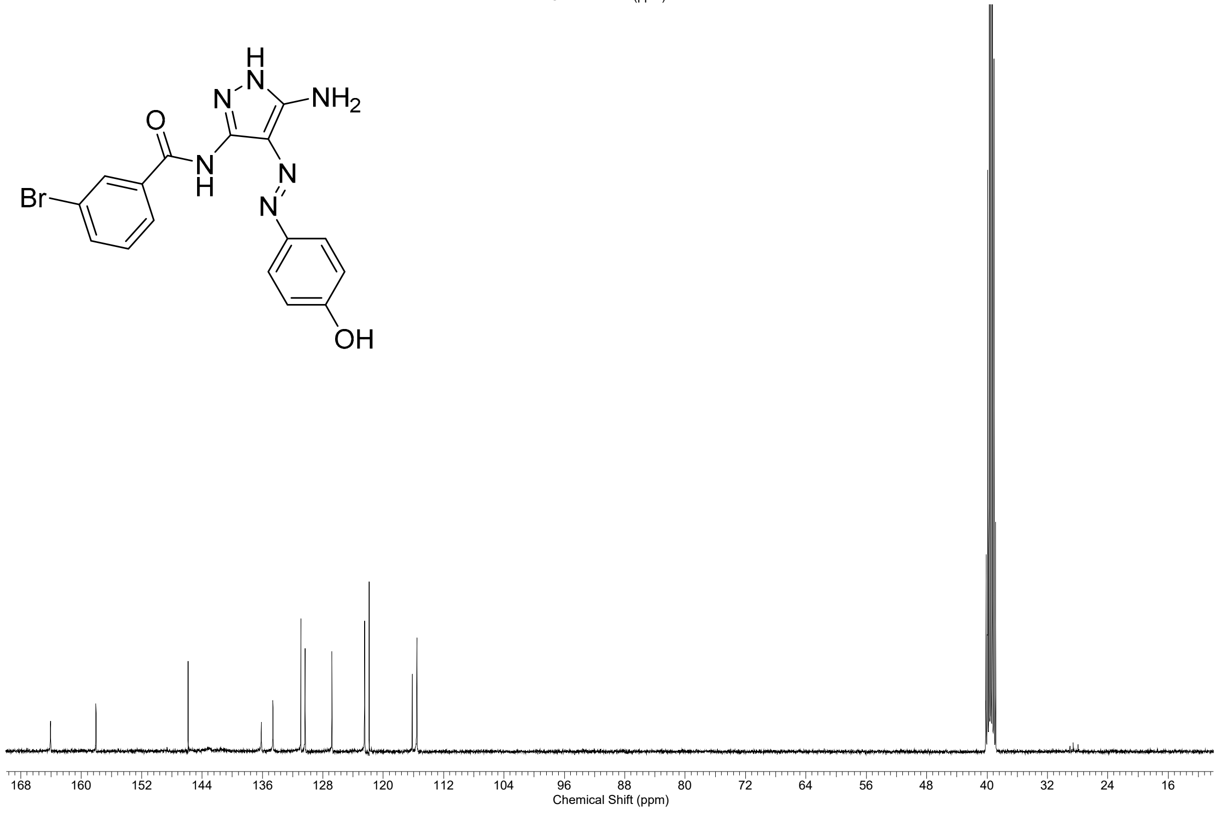

^{1}H and ^{13}C NMR spectra of 12c

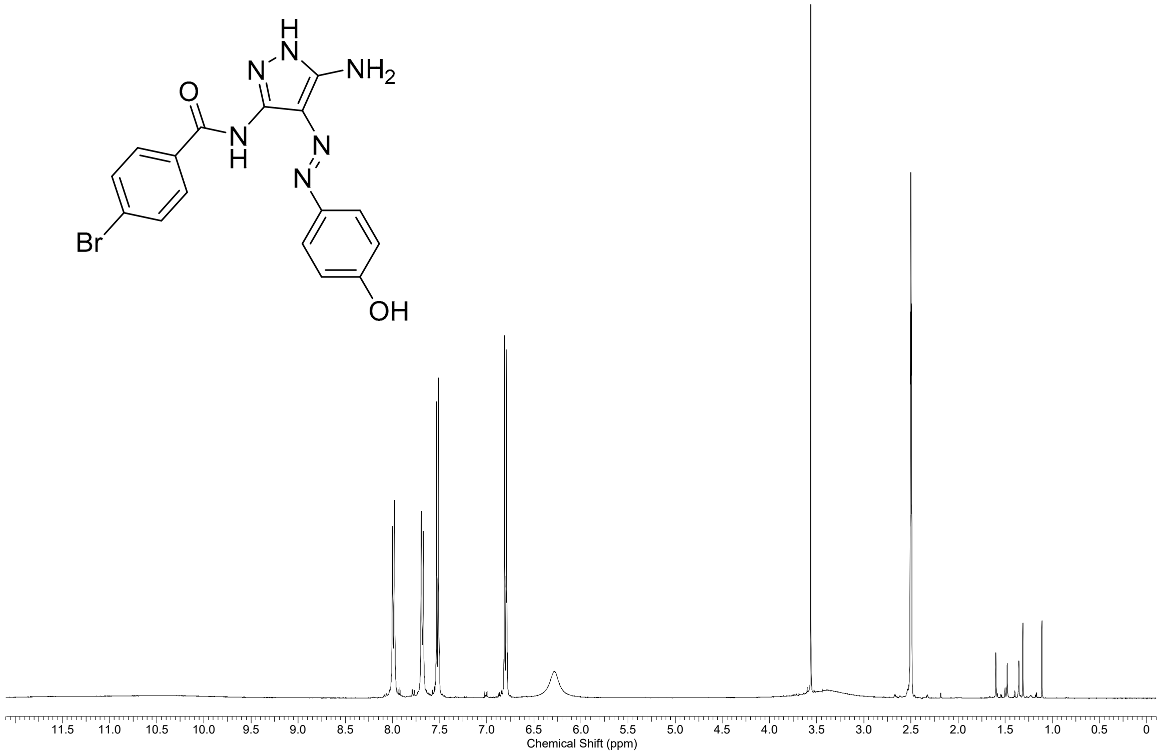

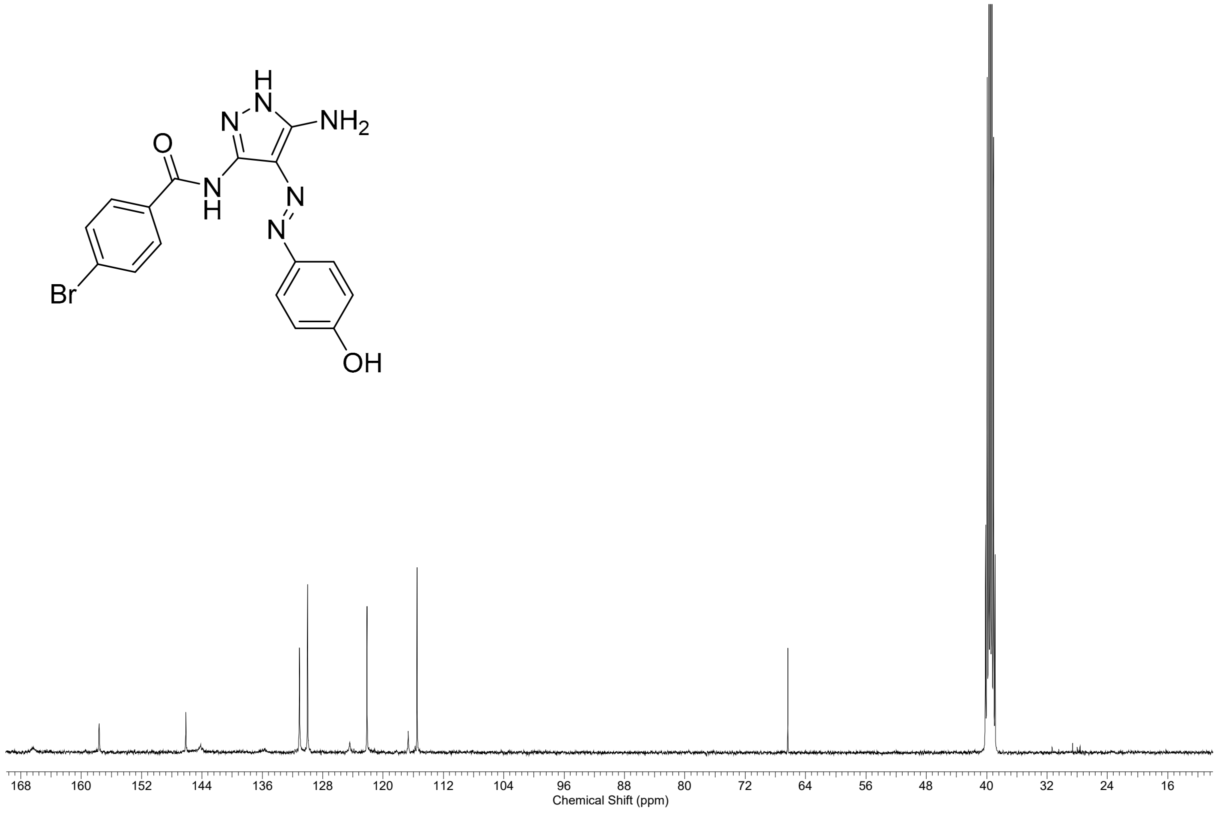

^{1}H and ^{13}C NMR spectra of 12d

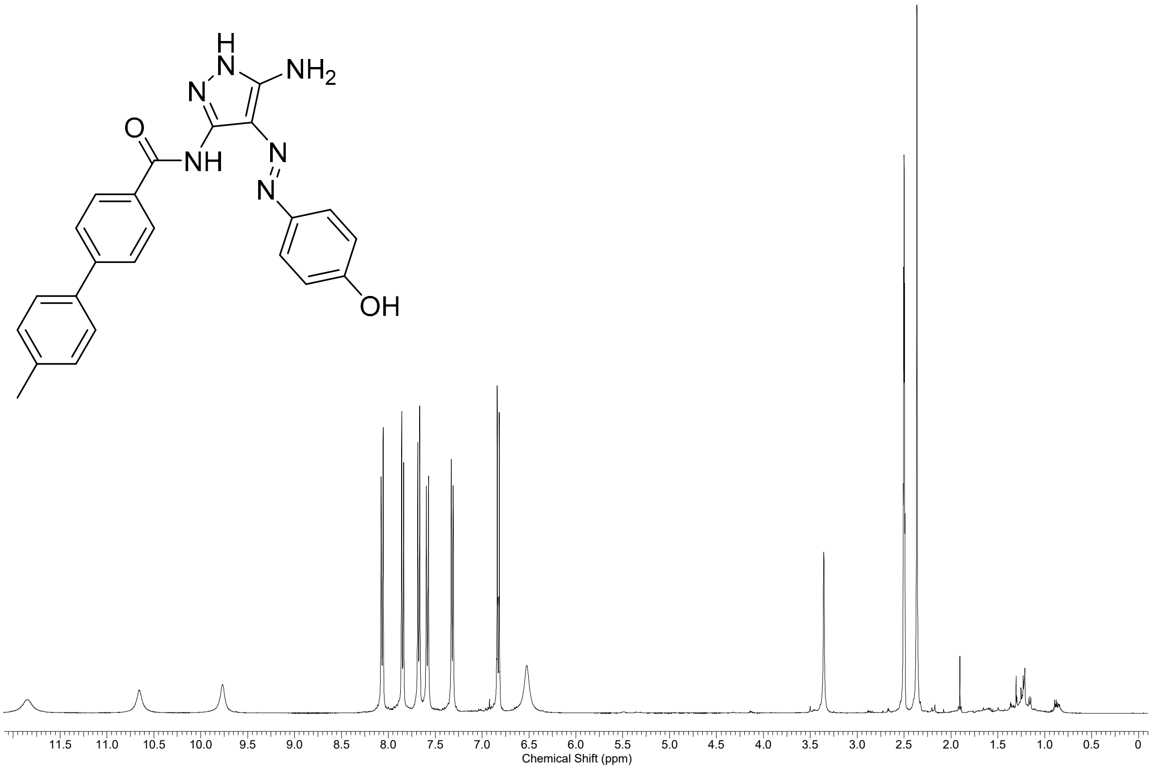

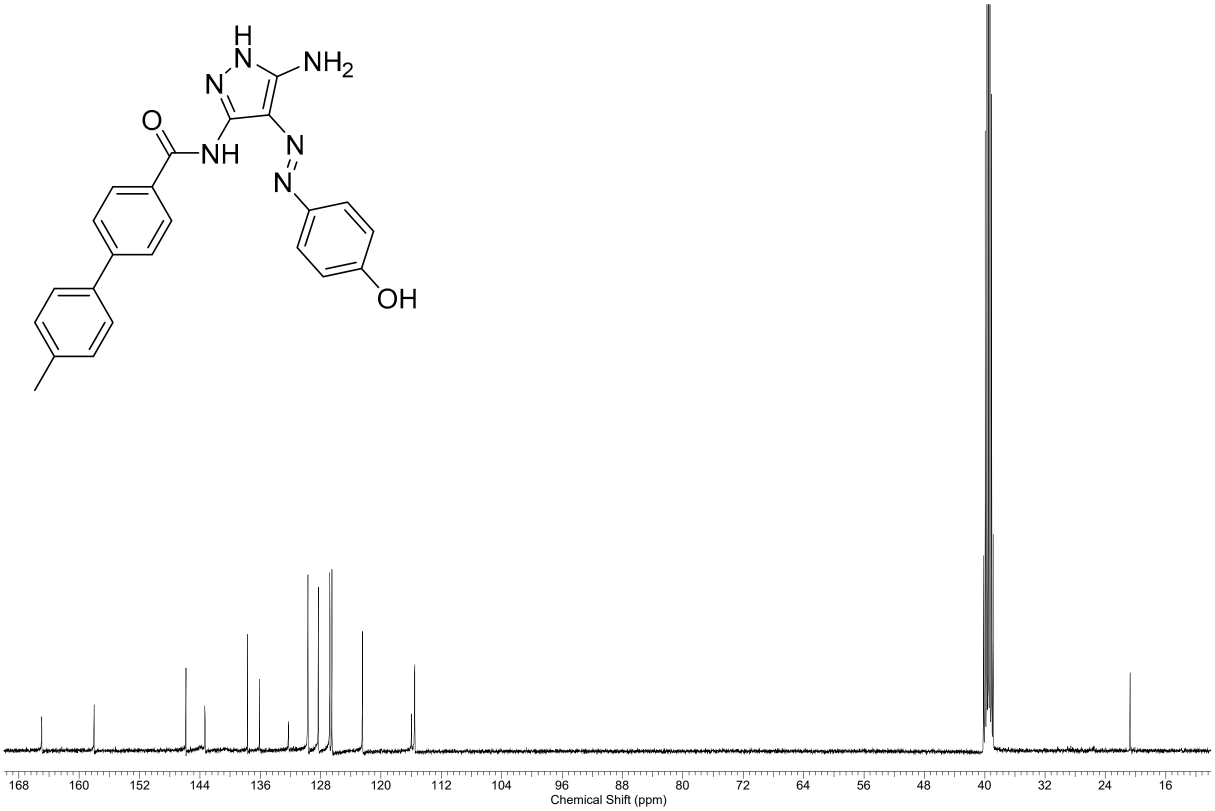

^{1}H and ^{13}C NMR spectra of 12e

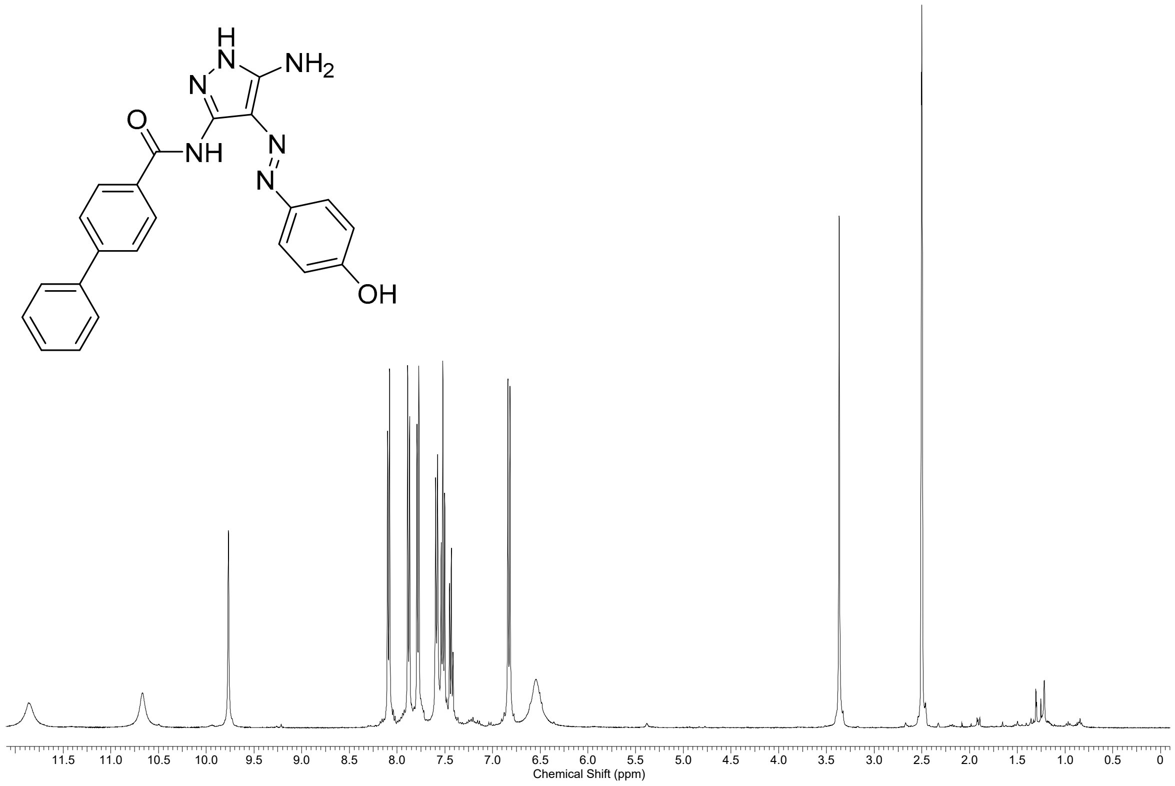

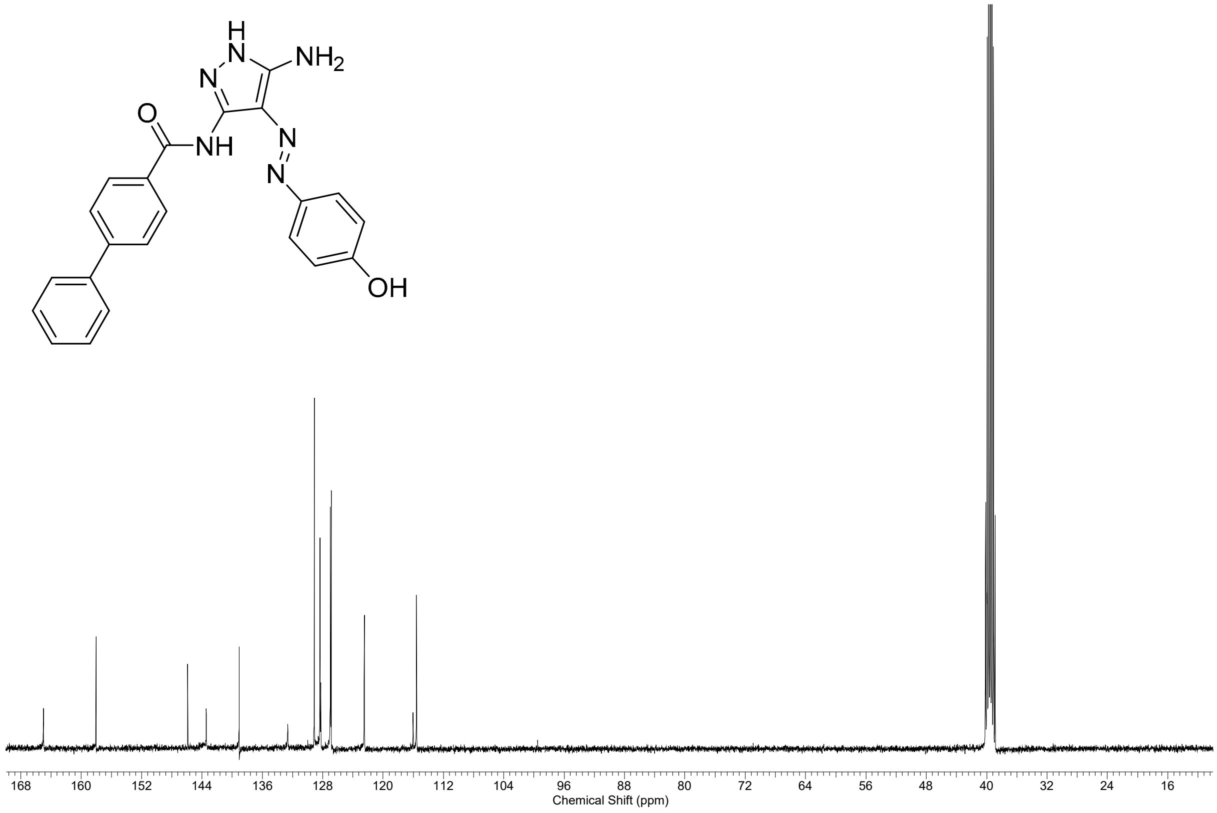

^{1}H and ^{13}C NMR spectra of 12f

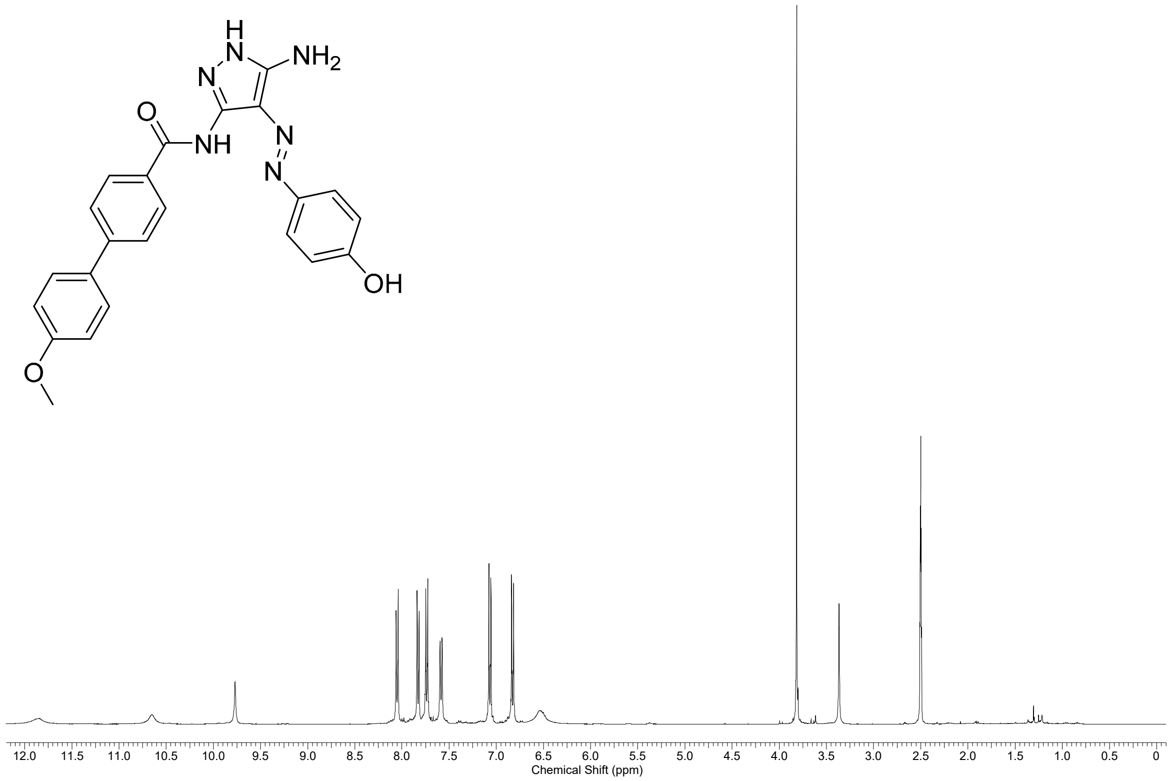

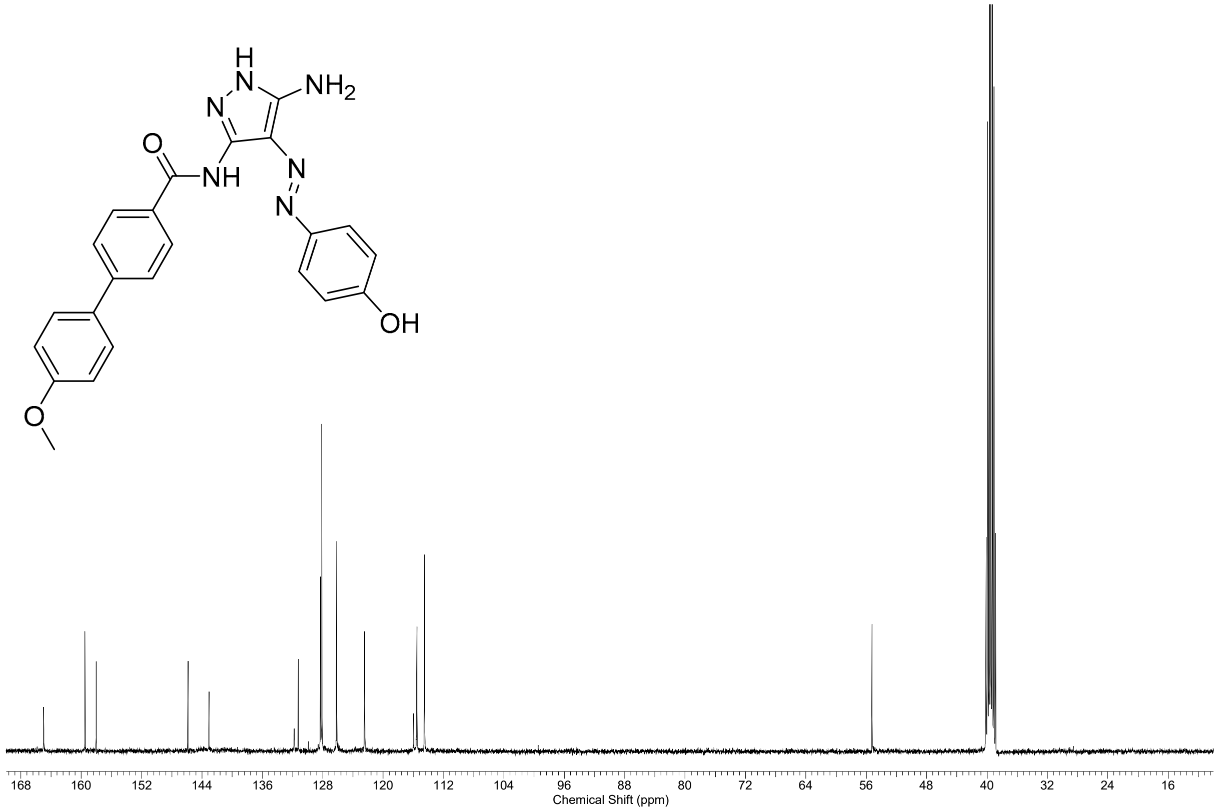

^{1}H and ^{13}C NMR spectra of 12g

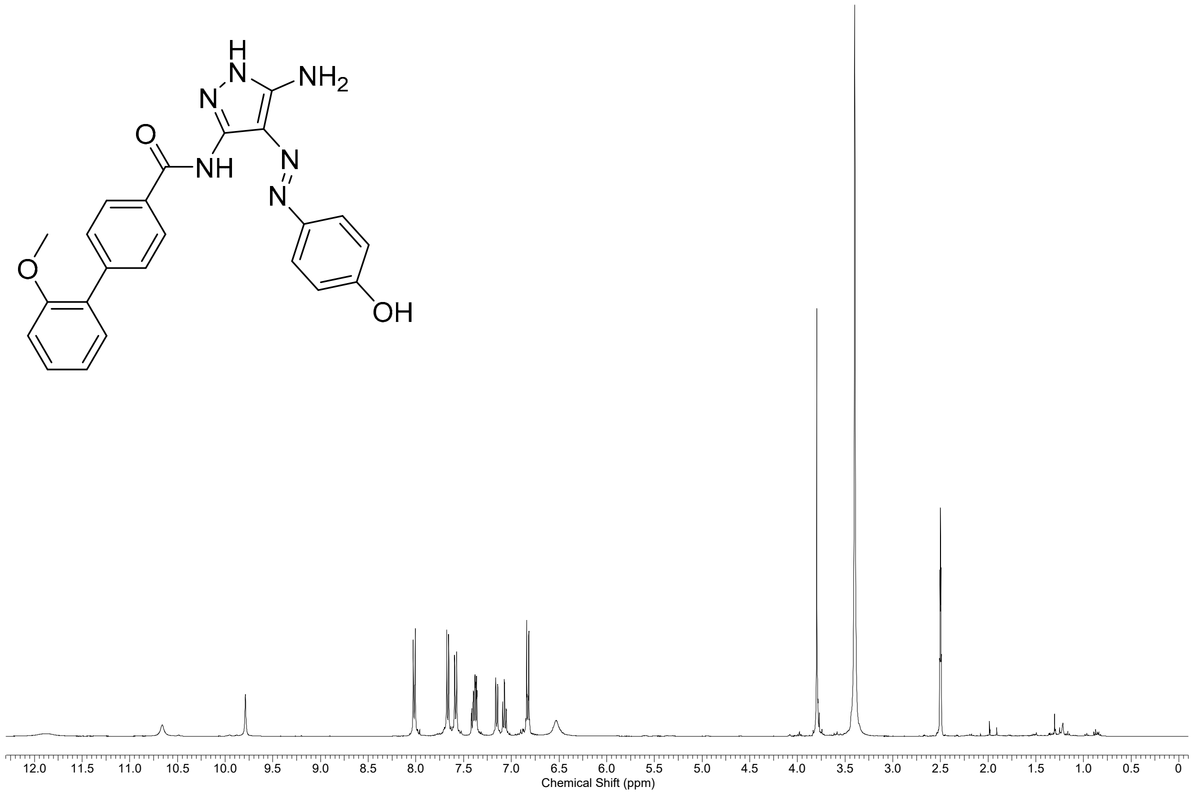

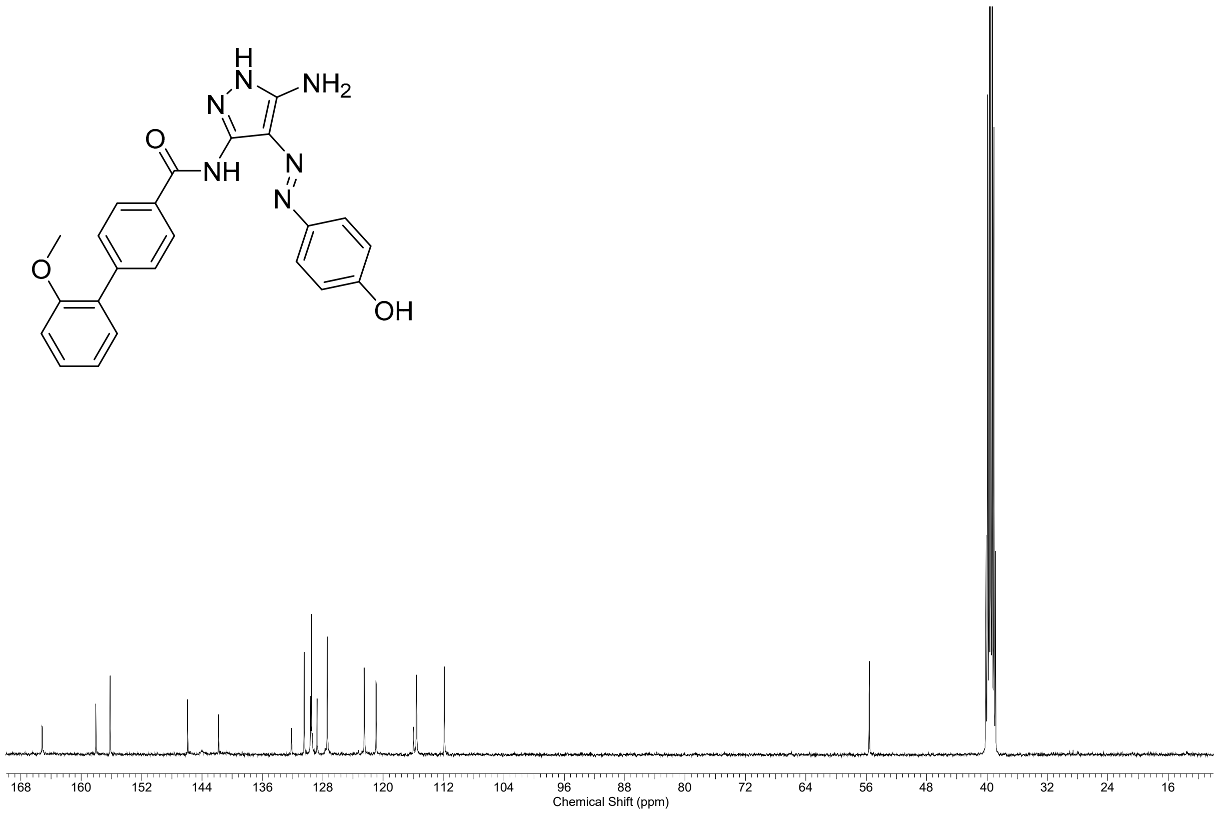

^{1}H and ^{13}C NMR spectra of 12h

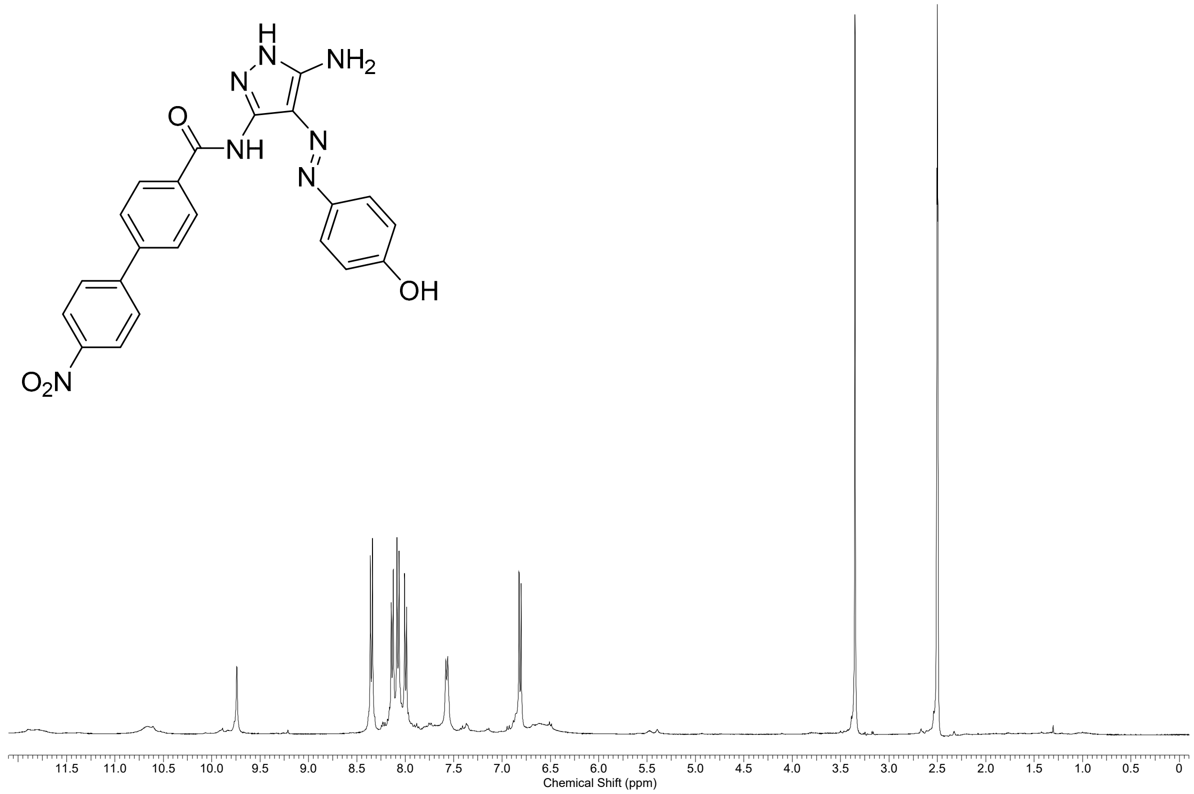

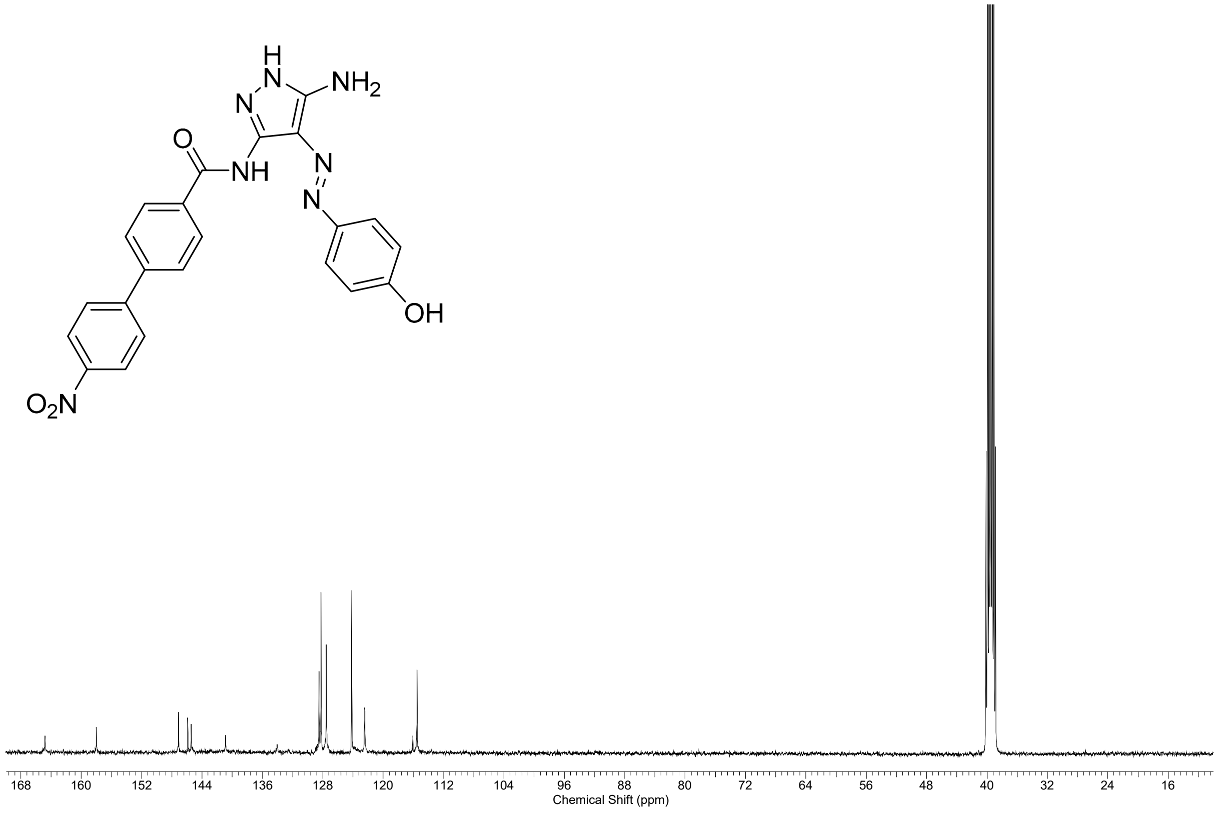

^{1}H and ^{13}C NMR spectra of 12i

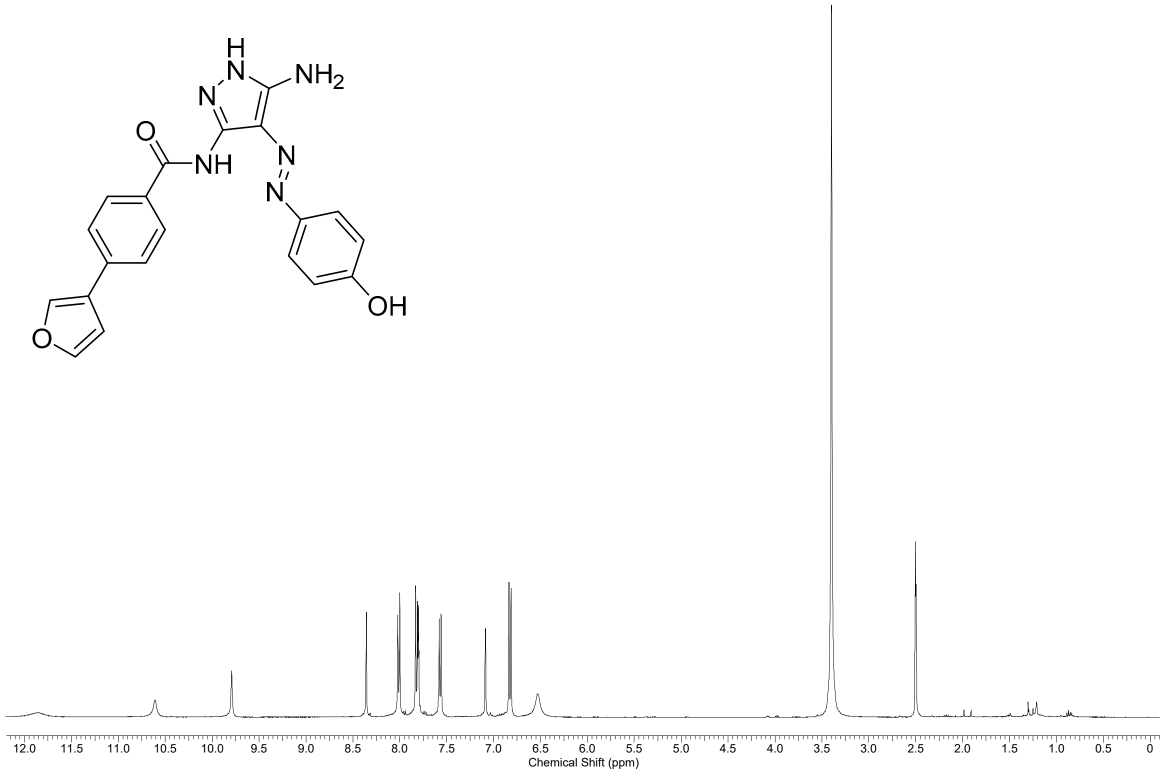

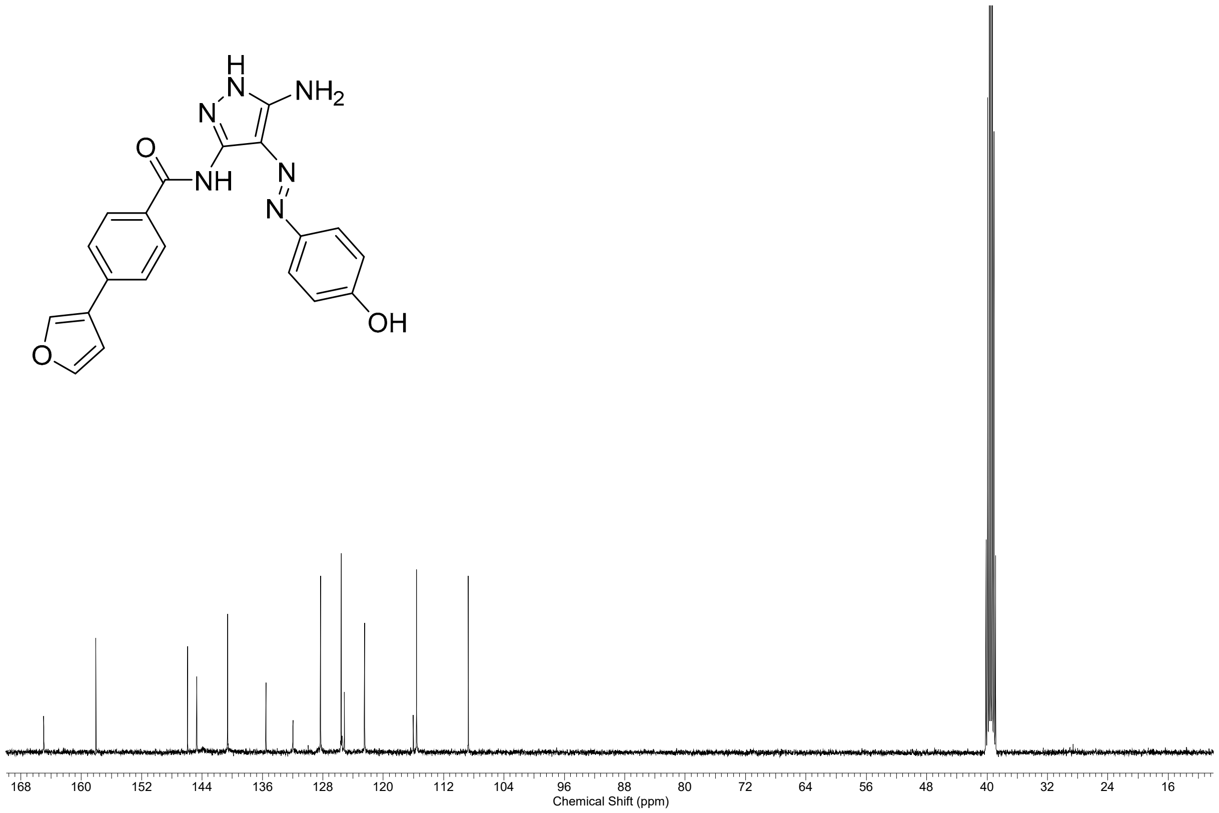

^{1}H and ^{13}C NMR spectra of 12j

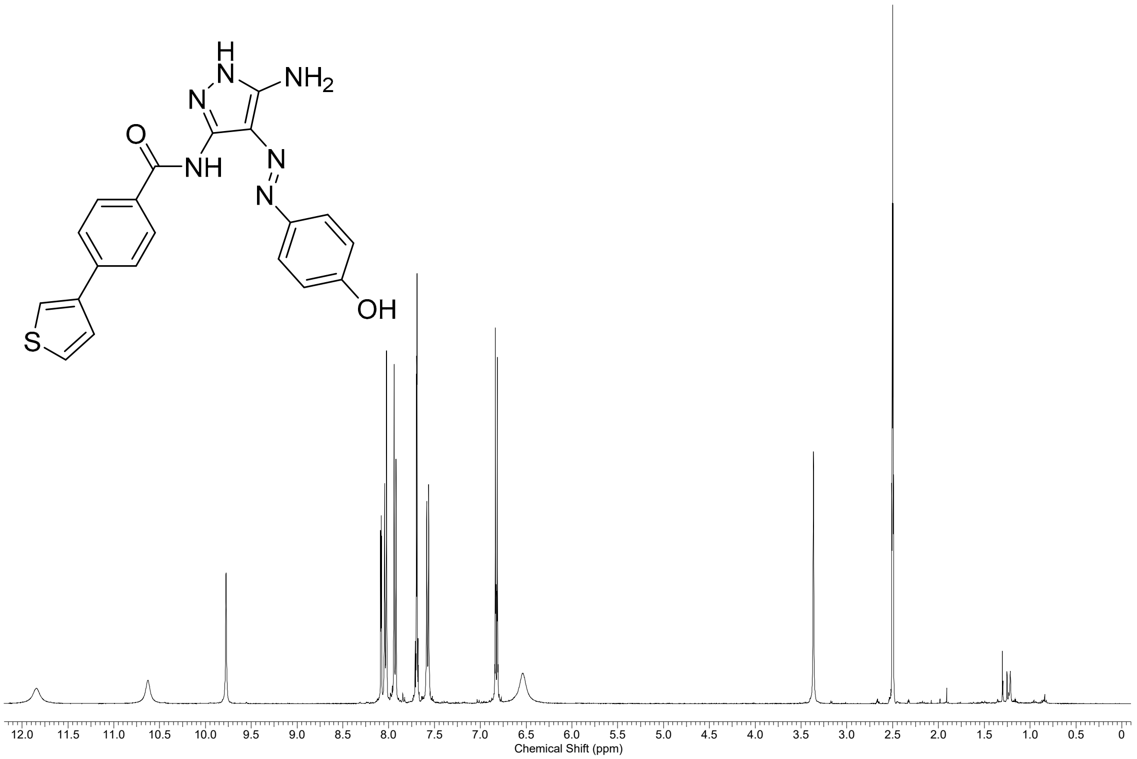

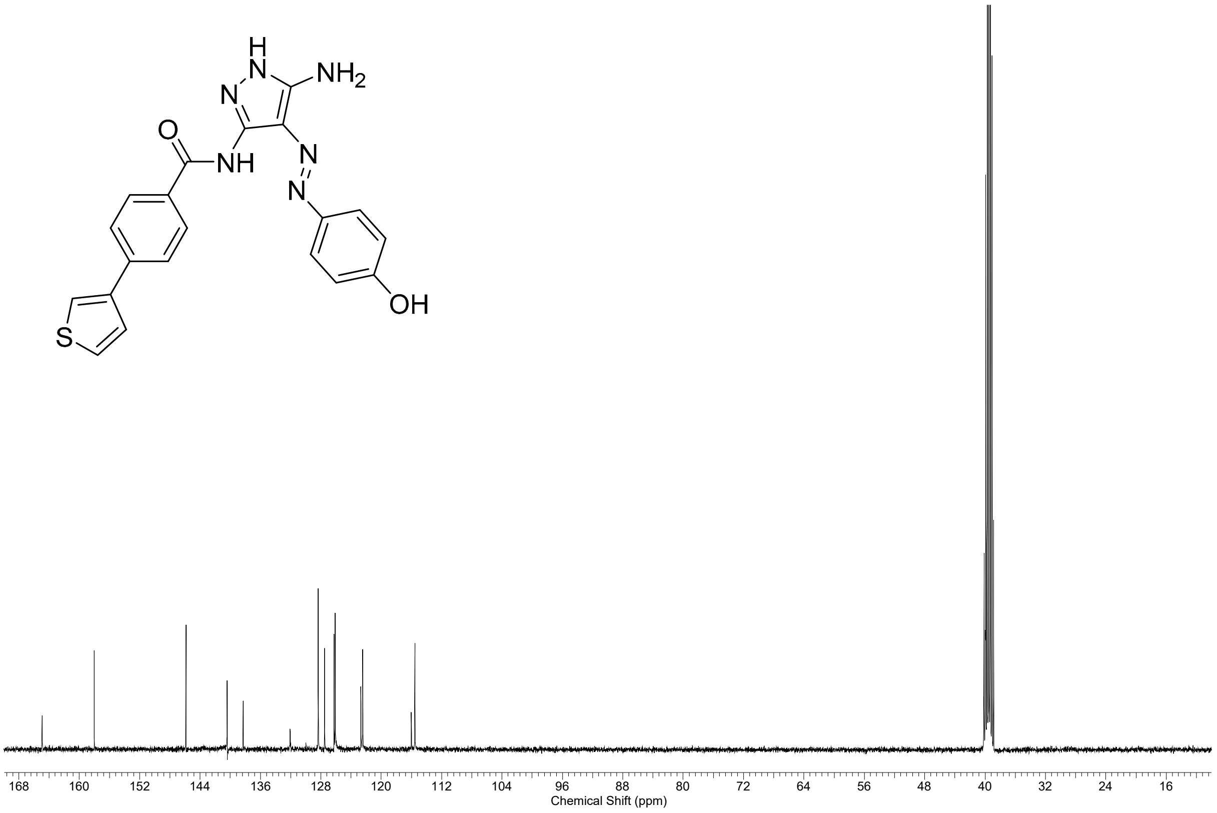

^{1}H and ^{13}C NMR spectra of 12k

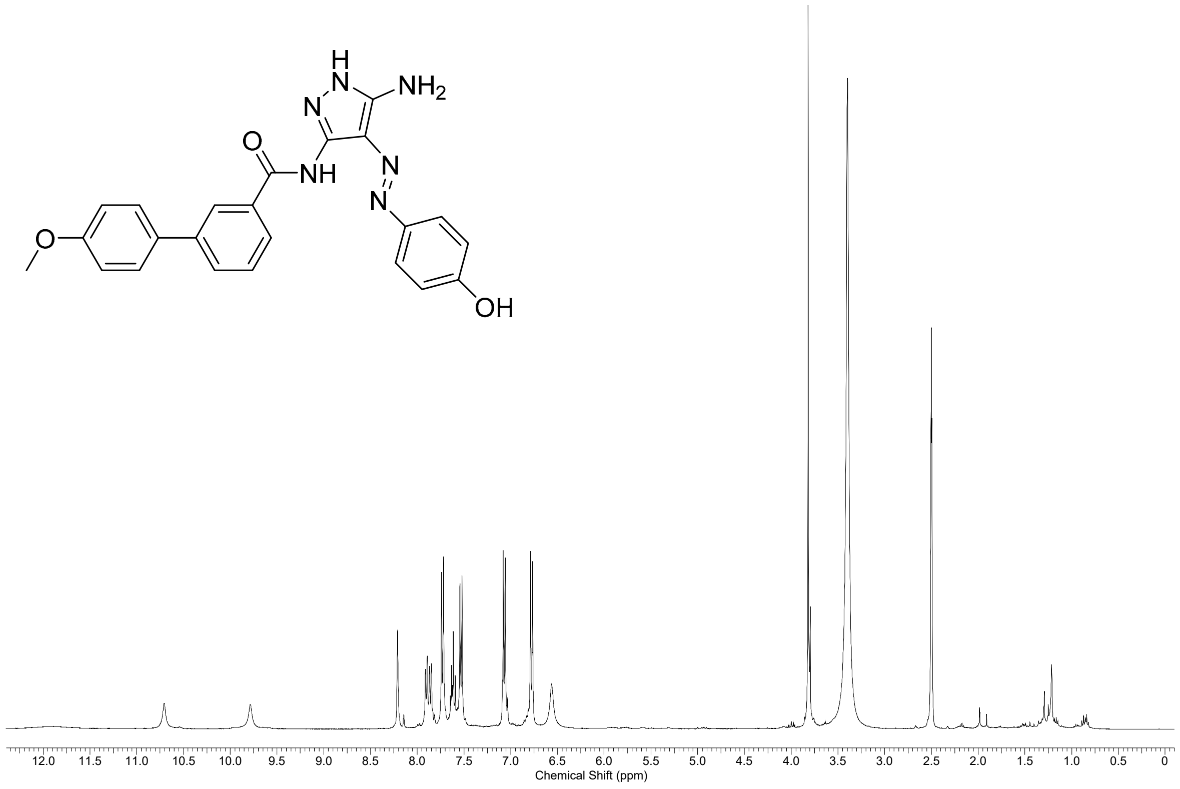

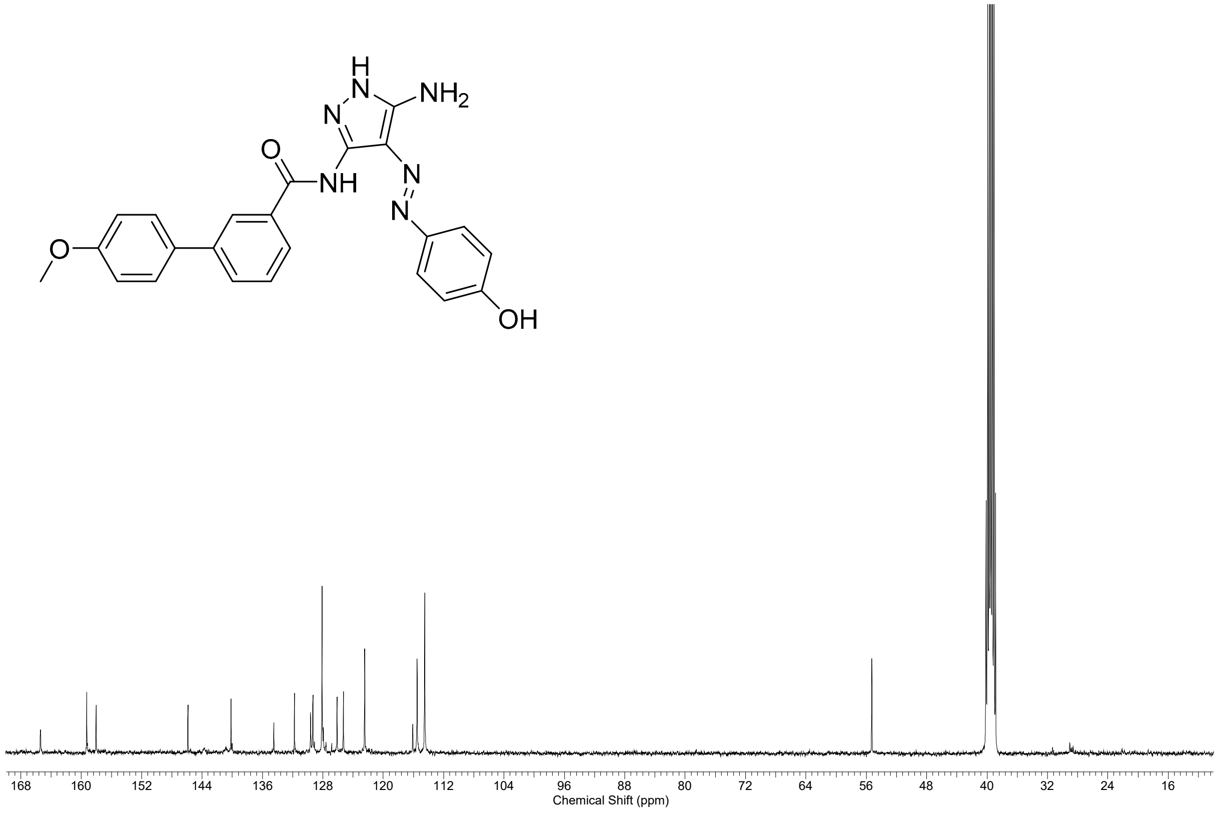

^{1}H and ^{13}C NMR spectra of 12l

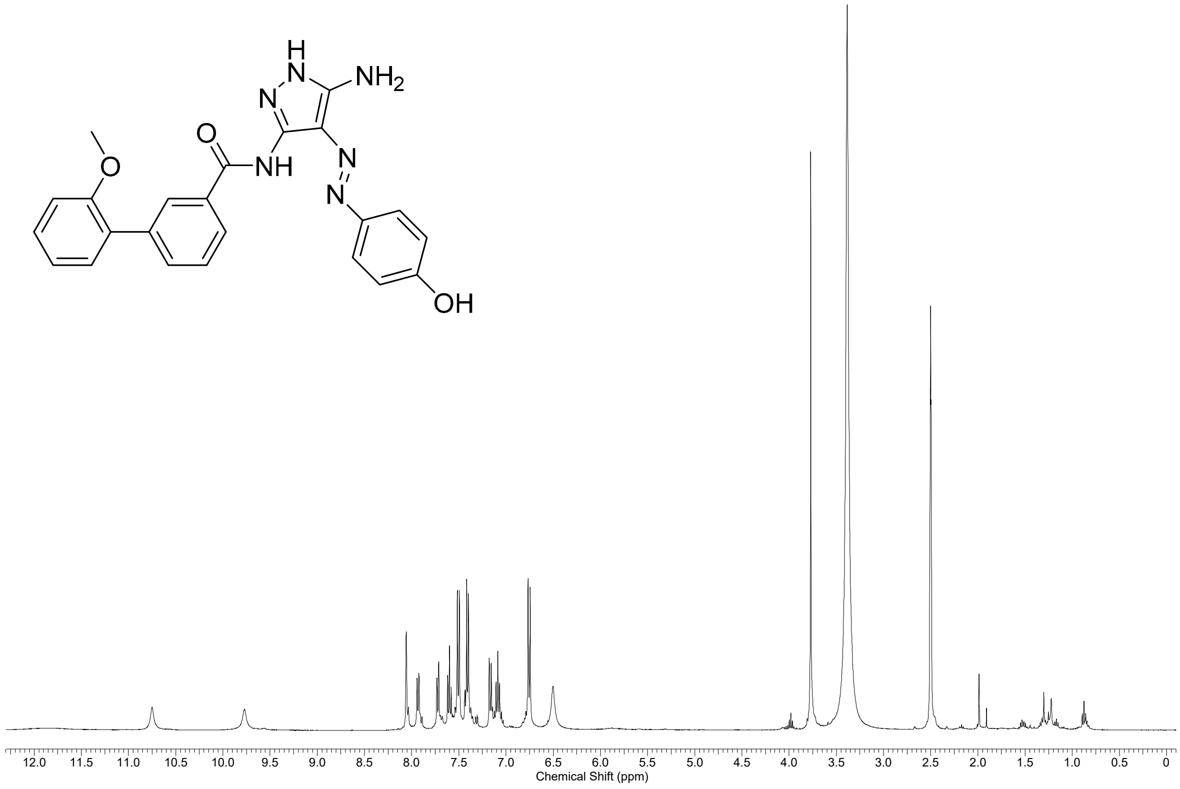

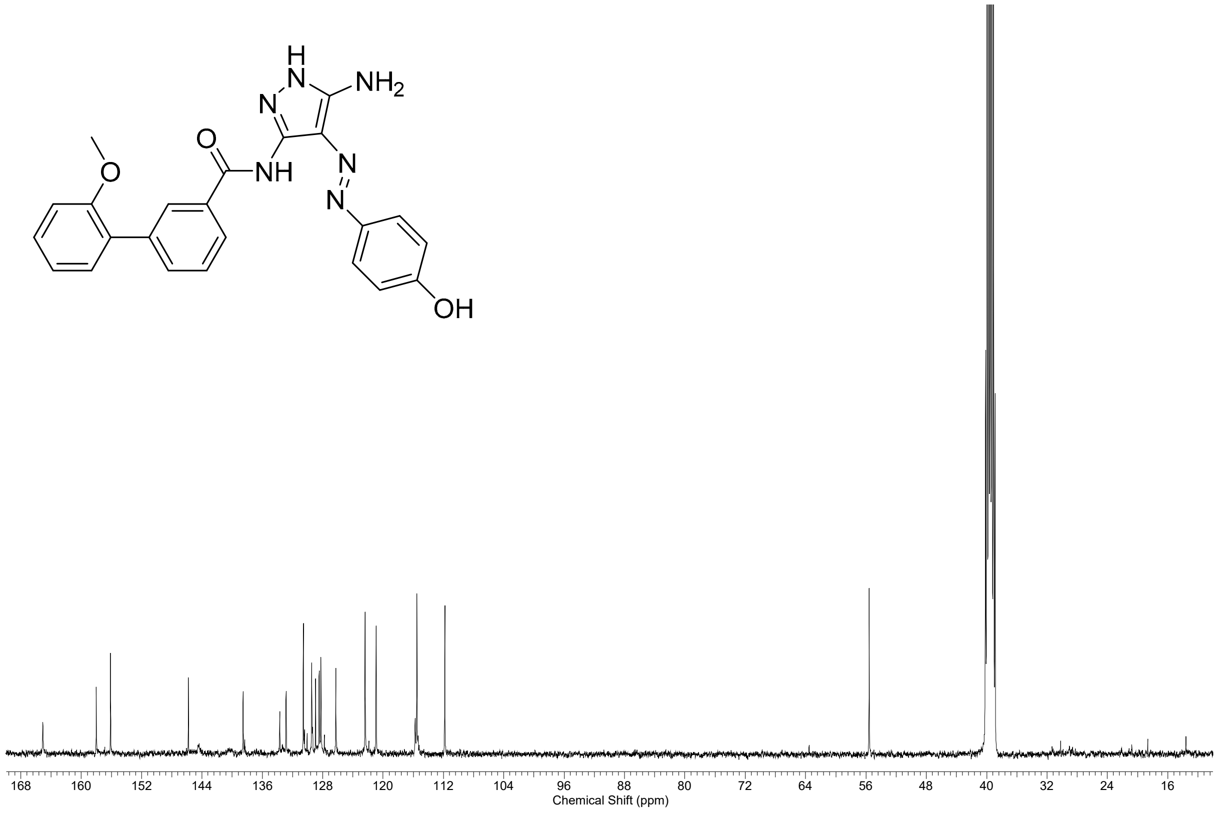

^{1}H and ^{13}C NMR spectra of 12m

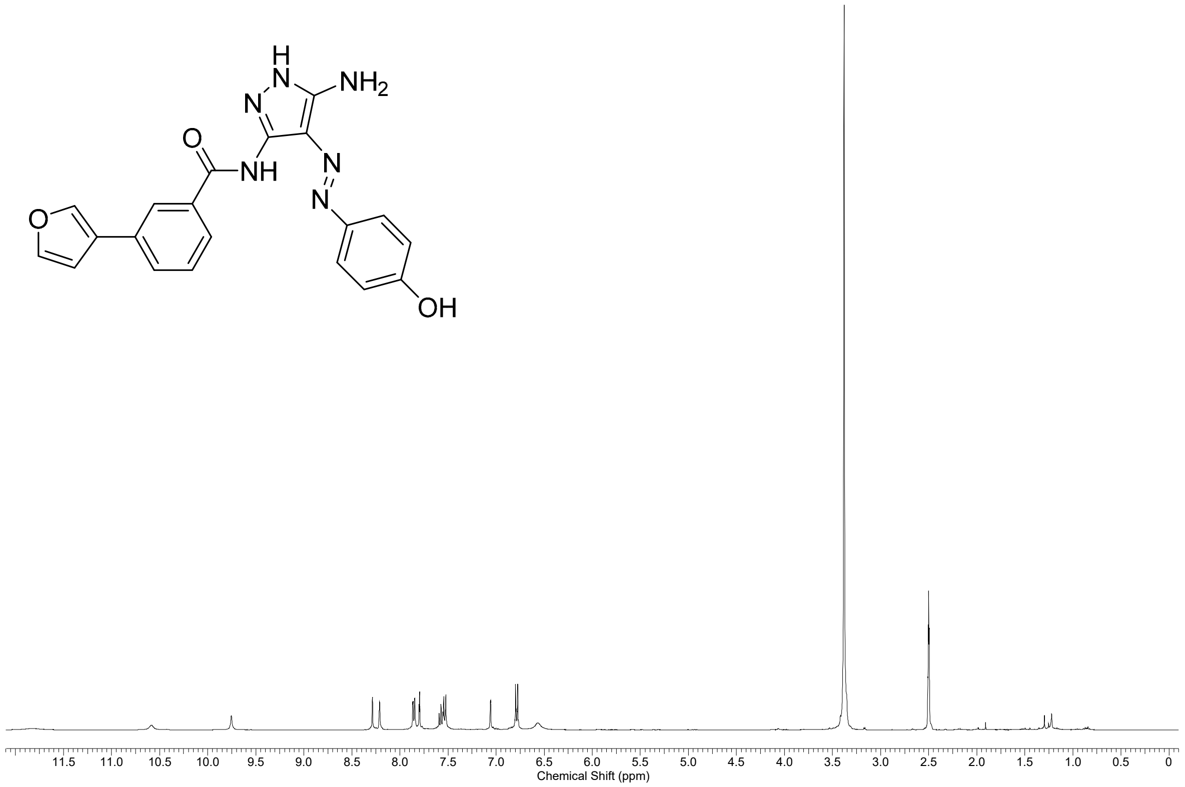

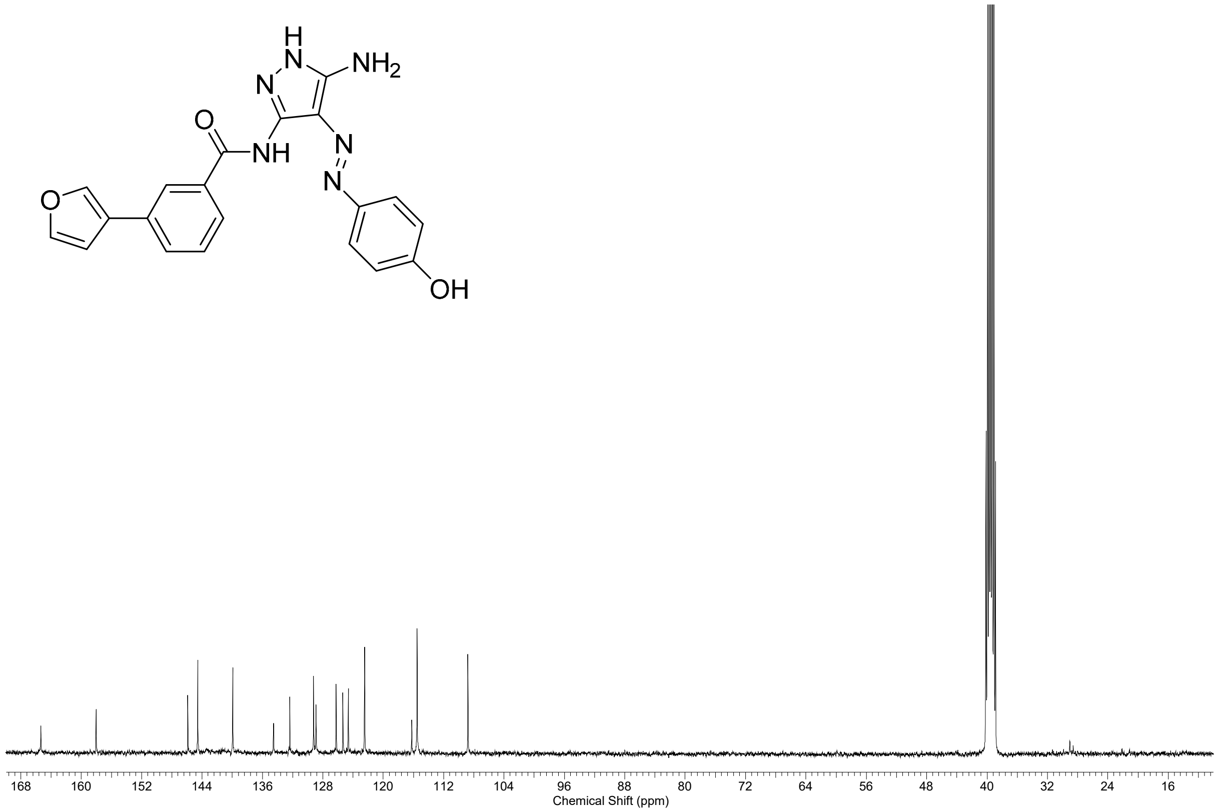

^{1}H and ^{13}C NMR spectra of 12n

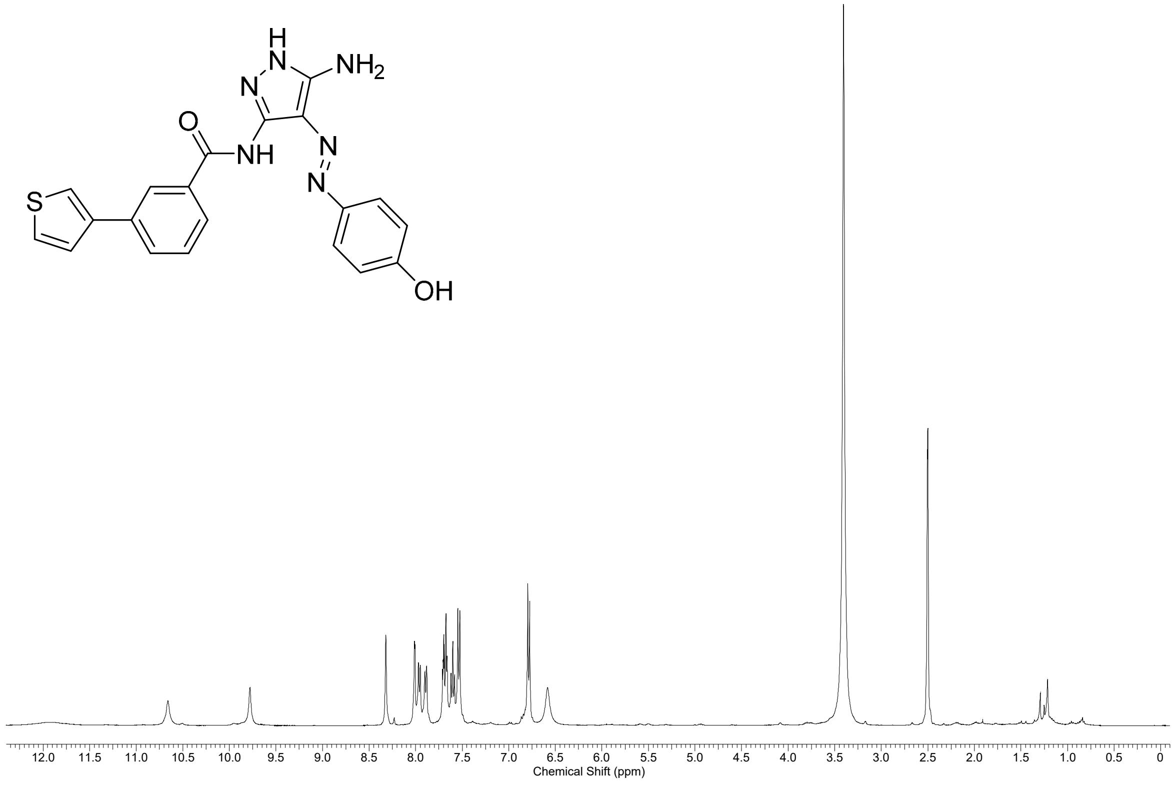

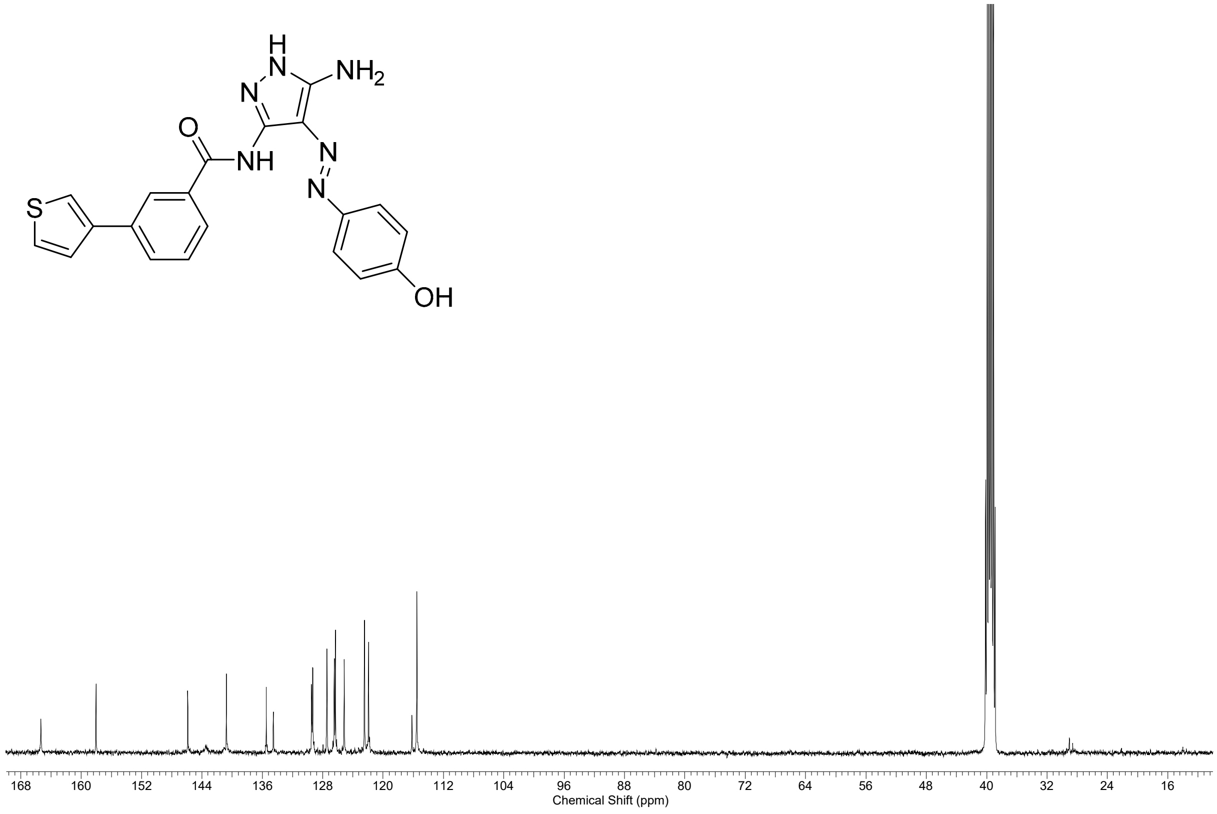

Single crystal X-ray analysis

The single crystal X-ray data of **3** (CCDC 1453321) and **5** (CCDC 1453320) were obtained using an Xcalibur2 diffractometer (Oxford Diffraction Ltd., UK) equipped with a Sapphire2 CCD detector, and with MoKα radiation (monochromator Enhance, Oxford Diffraction Ltd.) and ω-scan technique at 120 K. Additional details regarding structure determinations, such as crystal data and structure refinements, selected bond lengths and angles of covalent as well as non-covalent contacts are summarized below.

Data collection and reduction were performed by the CrysAlis software package.[1] The structure was solved by direct methods using SHELX[2] and refined on F^2 using a full-matrix least-squares procedure. All H-atoms were located from difference Fourier maps and refined using a riding model, with C−H = 0.95 Å $(CH)_{aromatic}$, 0.98 Å (CH_3), O–H = 0.84 Å and N–H = 0.88 Å $(NH)_{aromatic}$, and with $U_{iso}(H) = 1.2U_{eq}$(CH, NH, OH) and $1.5U_{eq}$ (CH_3). The H-atoms of the NH_2 groups were refined freely. Molecular graphics were drawn using DIAMOND.[3] Crystal data and structure refinements for **3** and **5** are given in Tables S1 and S2, sets of covalent and non-covalent bonding in Tables S3–S6, and parts of crystal structures are depicted in Figures S1–S4 (see below).

[1] Oxford Diffraction, CrysAlis RED and CrysAlis CCD Software (Version 1.171.33.52), Oxford Diffraction Ltd., Abingdon, Oxfordshire, UK.

[2] G.M. Sheldrick, A short history of SHELX, Acta Crystallogr., Sect. A 64 (2008) 112–122.

[3] K. Brandenburg DIAMOND, Release 4.1.1, Crystal Impact GbR, Bonn, Germany, 2015.

Table S1. Crystal data and structure refinement for **5**.

Empirical formula	$C_{14} H_{18} N_6 O_3$	
Formula weight	318.34	
Temperature	130(2) K	
Wavelength	0.71073 Å	
Crystal system	Monoclinic	
Space group	$P2_1/c$	
Unit cell dimensions	a = 6.2539(2) Å	α= 90°.
	b = 9.7519(4) Å	β= 94.809(3)°.
	c = 24.3894(9) Å	γ = 90°.
Volume	1482.20(10) $Å^3$	
Z	4	
Density (calculated)	1.427 Mg/m^3	
Absorption coefficient	0.105 mm^{-1}	
F(000)	672	
Crystal size	0.350 x 0.250 x 0.150 mm^3	
Theta range for data collection	3.269 to 24.990°.	
Index ranges	$-7 \leq h \leq 7, -9 \leq k \leq 11, -28 \leq l \leq 28$	
Reflections collected	12014	
Independent reflections	2610 [R(int) = 0.0287]	
Completeness to theta = 24.99°	99.9 %	
Absorption correction	Semi-empirical from equivalents	
Max. and min. transmission	1.000 and 0.985	
Refinement method	Full-matrix least-squares on F^2	
Data / restraints / parameters	2610 / 0 / 227	
Goodness-of-fit on F^2	1.009	
Final R indices [I>2sigma(I)]	$R1$ = 0.0312, w$R2$ = 0.0733	
R indices (all data)	$R1$ = 0.0445, w$R2$ = 0.0760	
Largest diff. peak and hole	0.201 and -0.180 e. $Å^{-3}$	

Table S2. Crystal data and structure refinement for **3**.

Empirical formula	$C_{19} H_{26} N_6 O_5$	
Formula weight	418.46	
Temperature	120(2) K	
Wavelength	0.71073 Å	
Crystal system	Triclinic	
Space group	*P*-1	
Unit cell dimensions	a = 6.1060(2) Å	α= 98.555(4)°.
	b = 11.9685(5) Å	β= 100.026(4)°.
	c = 14.7997(7) Å	γ = 97.021(3)°.
Volume	1040.93(8) Å^3	
Z	2	
Density (calculated)	1.335 Mg/m^3	
Absorption coefficient	0.099 mm^{-1}	
F(000)	444	
Crystal size	0.400 x 0.250 x 0.200 mm^3	
Theta range for data collection	3.061 to 24.999°.	
Index ranges	-7 ≤ h ≤ 7, -12 ≤ k ≤ 14, -17 ≤ l ≤ 17	
Reflections collected	9776	
Independent reflections	3667 [*R*(int) = 0.0246]	
Completeness to theta = 24.999°	99.7 %	
Absorption correction	Semi-empirical from equivalents	
Max. and min. transmission	1.000 and 0.866	
Refinement method	Full-matrix least-squares on F^2	
Data / restraints / parameters	3667 / 0 / 293	
Goodness-of-fit on F^2	1.061	
Final *R* indices [I>2sigma(I)]	*R*1 = 0.0359, w*R*2 = 0.0987	
R indices (all data)	*R*1 = 0.0508, w*R*2 = 0.1021	
Largest diff. peak and hole	0.187 and -0.190 e. Å^{-3}	

Table S3. Bond lengths [Å] and angles [°] for **5**.

O(1)-C(7)	1.3663(15)
O(1)-H(1A)	0.8400
N(1)-C(1)	1.3744(16)
N(1)-C(10)	1.3933(16)
N(1)-N(2)	1.4096(15)
C(1)-N(6)	1.3713(17)
C(1)-C(2)	1.3752(18)
N(2)-C(3)	1.3261(16)
O(2)-C(10)	1.2097(16)
C(2)-N(3)	1.3805(16)
C(2)-C(3)	1.4322(18)
O(3)-C(10)	1.3226(16)
O(3)-C(11)	1.5033(15)
N(3)-N(4)	1.2781(15)
C(3)-N(5)	1.3446(17)
N(4)-C(4)	1.4190(17)
C(4)-C(9)	1.3896(19)
C(4)-C(5)	1.3921(19)
N(5)-H(5D)	0.857(17)

N(5)-H(5C)	0.908(18)
C(5)-C(6)	1.3836(18)
C(5)-H(5A)	0.9500
N(6)-H(6D)	0.898(18)
N(6)-H(6C)	0.881(17)
C(6)-C(7)	1.383(2)
C(6)-H(6A)	0.9500
C(7)-C(8)	1.3856(19)
C(8)-C(9)	1.3793(18)
C(8)-H(8A)	0.9500
C(9)-H(9A)	0.9500
C(11)-C(14)	1.5139(19)
C(11)-C(13)	1.5144(19)
C(11)-C(12)	1.5136(19)
C(12)-H(12A)	0.9800
C(12)-H(12B)	0.9800
C(12)-H(12C)	0.9800
C(13)-H(13A)	0.9800
C(13)-H(13B)	0.9800
C(13)-H(13C)	0.9800
C(14)-H(14A)	0.9800
C(14)-H(14B)	0.9800
C(14)-H(14C)	0.9800
C(7)-O(1)-H(1A)	109.5
C(1)-N(1)-C(10)	125.79(11)
C(1)-N(1)-N(2)	111.54(10)
C(10)-N(1)-N(2)	122.09(10)
N(6)-C(1)-N(1)	123.48(12)
N(6)-C(1)-C(2)	129.73(12)
N(1)-C(1)-C(2)	106.78(11)
C(3)-N(2)-N(1)	103.99(10)
C(1)-C(2)-N(3)	122.71(12)
C(1)-C(2)-C(3)	105.50(11)
N(3)-C(2)-C(3)	131.69(12)
C(10)-O(3)-C(11)	119.47(10)
N(4)-N(3)-C(2)	114.18(11)
N(2)-C(3)-N(5)	122.16(12)
N(2)-C(3)-C(2)	112.14(11)
N(5)-C(3)-C(2)	125.70(12)
N(3)-N(4)-C(4)	113.80(11)
C(9)-C(4)-C(5)	119.13(12)
C(9)-C(4)-N(4)	124.42(12)
C(5)-C(4)-N(4)	116.44(12)
C(3)-N(5)-H(5D)	117.6(11)
C(3)-N(5)-H(5C)	120.4(10)
H(5D)-N(5)-H(5C)	120.1(15)
C(6)-C(5)-C(4)	120.52(13)
C(6)-C(5)-H(5A)	119.7
C(4)-C(5)-H(5A)	119.7
C(1)-N(6)-H(6D)	113.1(11)
C(1)-N(6)-H(6C)	111.4(11)
H(6D)-N(6)-H(6C)	114.6(15)
C(7)-C(6)-C(5)	119.80(13)
C(7)-C(6)-H(6A)	120.1
C(5)-C(6)-H(6A)	120.1
O(1)-C(7)-C(6)	117.73(12)

O(1)-C(7)-C(8)	122.26(13)
C(6)-C(7)-C(8)	120.01(12)
C(9)-C(8)-C(7)	120.21(13)
C(9)-C(8)-H(8A)	119.9
C(7)-C(8)-H(8A)	119.9
C(8)-C(9)-C(4)	120.31(13)
C(8)-C(9)-H(9A)	119.8
C(4)-C(9)-H(9A)	119.8
O(2)-C(10)-O(3)	127.61(12)
O(2)-C(10)-N(1)	121.00(12)
O(3)-C(10)-N(1)	111.37(11)
O(3)-C(11)-C(14)	111.21(11)
O(3)-C(11)-C(13)	101.98(10)
C(14)-C(11)-C(13)	110.21(12)
O(3)-C(11)-C(12)	108.10(10)
C(14)-C(11)-C(12)	113.04(12)
C(13)-C(11)-C(12)	111.75(12)
C(11)-C(12)-H(12A)	109.5
C(11)-C(12)-H(12B)	109.5
H(12A)-C(12)-H(12B)	109.5
C(11)-C(12)-H(12C)	109.5
H(12A)-C(12)-H(12C)	109.5
H(12B)-C(12)-H(12C)	109.5
C(11)-C(13)-H(13A)	109.5
C(11)-C(13)-H(13B)	109.5
H(13A)-C(13)-H(13B)	109.5
C(11)-C(13)-H(13C)	109.5
H(13A)-C(13)-H(13C)	109.5
H(13B)-C(13)-H(13C)	109.5
C(11)-C(14)-H(14A)	109.5
C(11)-C(14)-H(14B)	109.5
H(14A)-C(14)-H(14B)	109.5
C(11)-C(14)-H(14C)	109.5
H(14A)-C(14)-H(14C)	109.5
H(14B)-C(14)-H(14C)	109.5

Table S4. Bond lengths [Å] and angles [°] for **3**.

O(1)-C(10)	1.322(2)
O(1)-C(11)	1.4821(19)
O(2)-C(10)	1.207(2)
O(3)-C(15)	1.358(2)
O(3)-C(7)	1.4105(19)
O(4)-C(15)	1.1950(19)
O(5)-C(15)	1.323(2)
O(5)-C(16)	1.4892(19)
N(1)-C(3)	1.374(2)
N(1)-C(10)	1.386(2)
N(1)-N(2)	1.4109(19)
N(2)-C(1)	1.318(2)
N(3)-N(4)	1.2773(19)
N(3)-C(2)	1.374(2)
N(4)-C(4)	1.418(2)
N(5)-C(1)	1.355(2)
N(5)-H(5B)	0.90(2)
N(5)-H(5A)	0.87(2)
N(6)-C(3)	1.342(2)
N(6)-H(6B)	0.88(2)
N(6)-H(6A)	0.88(2)
C(1)-C(2)	1.437(2)
C(2)-C(3)	1.382(2)
C(4)-C(9)	1.387(2)
C(4)-C(5)	1.395(2)
C(5)-C(6)	1.377(2)
C(5)-H(5C)	0.9500
C(6)-C(7)	1.382(2)
C(6)-H(6C)	0.9500
C(7)-C(8)	1.375(2)
C(8)-C(9)	1.381(2)
C(8)-H(8A)	0.9500
C(9)-H(9A)	0.9500
C(11)-C(12)	1.505(3)
C(11)-C(14)	1.509(2)
C(11)-C(13)	1.516(2)
C(12)-H(12A)	0.9800
C(12)-H(12B)	0.9800
C(12)-H(12C)	0.9800
C(13)-H(13A)	0.9800
C(13)-H(13B)	0.9800
C(13)-H(13C)	0.9800
C(14)-H(14A)	0.9800
C(14)-H(14B)	0.9800
C(14)-H(14C)	0.9800
C(16)-C(19)	1.512(2)
C(16)-C(17)	1.509(3)
C(16)-C(18)	1.519(3)
C(17)-H(17A)	0.9800
C(17)-H(17B)	0.9800
C(17)-H(17C)	0.9800
C(18)-H(18A)	0.9800
C(18)-H(18B)	0.9800
C(18)-H(18C)	0.9800

C(19)-H(19A)	0.9800
C(19)-H(19B)	0.9800
C(19)-H(19C)	0.9800
C(10)-O(1)-C(11)	119.67(12)
C(15)-O(3)-C(7)	115.27(12)
C(15)-O(5)-C(16)	119.13(12)
C(3)-N(1)-C(10)	126.16(14)
C(3)-N(1)-N(2)	111.78(12)
C(10)-N(1)-N(2)	121.79(13)
C(1)-N(2)-N(1)	104.20(12)
N(4)-N(3)-C(2)	114.15(13)
N(3)-N(4)-C(4)	114.32(13)
C(1)-N(5)-H(5B)	121.5(13)
C(1)-N(5)-H(5A)	116.7(12)
H(5B)-N(5)-H(5A)	114.8(18)
C(3)-N(6)-H(6B)	118.4(13)
C(3)-N(6)-H(6A)	113.6(13)
H(6B)-N(6)-H(6A)	117.0(19)
N(2)-C(1)-N(5)	122.19(15)
N(2)-C(1)-C(2)	112.17(14)
N(5)-C(1)-C(2)	125.60(15)
N(3)-C(2)-C(3)	122.59(14)
N(3)-C(2)-C(1)	131.90(14)
C(3)-C(2)-C(1)	105.48(14)
N(6)-C(3)-N(1)	124.26(15)
N(6)-C(3)-C(2)	129.34(15)
N(1)-C(3)-C(2)	106.37(14)
C(9)-C(4)-C(5)	119.42(15)
C(9)-C(4)-N(4)	115.42(14)
C(5)-C(4)-N(4)	125.12(14)
C(6)-C(5)-C(4)	119.85(15)
C(6)-C(5)-H(5C)	120.1
C(4)-C(5)-H(5C)	120.1
C(5)-C(6)-C(7)	119.58(15)
C(5)-C(6)-H(6C)	120.2
C(7)-C(6)-H(6C)	120.2
C(8)-C(7)-C(6)	121.45(15)
C(8)-C(7)-O(3)	117.53(14)
C(6)-C(7)-O(3)	120.98(14)
C(7)-C(8)-C(9)	118.83(15)
C(7)-C(8)-H(8A)	120.6
C(9)-C(8)-H(8A)	120.6
C(8)-C(9)-C(4)	120.81(15)
C(8)-C(9)-H(9A)	119.6
C(4)-C(9)-H(9A)	119.6
O(2)-C(10)-O(1)	127.56(15)
O(2)-C(10)-N(1)	121.91(15)
O(1)-C(10)-N(1)	110.53(14)
O(1)-C(11)-C(12)	107.60(14)
O(1)-C(11)-C(14)	111.64(13)
C(12)-C(11)-C(14)	113.01(16)
O(1)-C(11)-C(13)	101.38(13)
C(12)-C(11)-C(13)	111.66(16)
C(14)-C(11)-C(13)	110.92(15)
C(11)-C(12)-H(12A)	109.5

C(11)-C(12)-H(12B)	109.5
H(12A)-C(12)-H(12B)	109.5
C(11)-C(12)-H(12C)	109.5
H(12A)-C(12)-H(12C)	109.5
H(12B)-C(12)-H(12C)	109.5
C(11)-C(13)-H(13A)	109.5
C(11)-C(13)-H(13B)	109.5
H(13A)-C(13)-H(13B)	109.5
C(11)-C(13)-H(13C)	109.5
H(13A)-C(13)-H(13C)	109.5
H(13B)-C(13)-H(13C)	109.5
C(11)-C(14)-H(14A)	109.5
C(11)-C(14)-H(14B)	109.5
H(14A)-C(14)-H(14B)	109.5
C(11)-C(14)-H(14C)	109.5
H(14A)-C(14)-H(14C)	109.5
H(14B)-C(14)-H(14C)	109.5
O(4)-C(15)-O(5)	128.79(15)
O(4)-C(15)-O(3)	124.97(15)
O(5)-C(15)-O(3)	106.24(13)
O(5)-C(16)-C(19)	102.13(13)
O(5)-C(16)-C(17)	109.25(14)
C(19)-C(16)-C(17)	111.37(15)
O(5)-C(16)-C(18)	109.84(13)
C(19)-C(16)-C(18)	110.43(15)
C(17)-C(16)-C(18)	113.25(15)
C(16)-C(17)-H(17A)	109.5
C(16)-C(17)-H(17B)	109.5
H(17A)-C(17)-H(17B)	109.5
C(16)-C(17)-H(17C)	109.5
H(17A)-C(17)-H(17C)	109.5
H(17B)-C(17)-H(17C)	109.5
C(16)-C(18)-H(18A)	109.5
C(16)-C(18)-H(18B)	109.5
H(18A)-C(18)-H(18B)	109.5
C(16)-C(18)-H(18C)	109.5
H(18A)-C(18)-H(18C)	109.5
H(18B)-C(18)-H(18C)	109.5
C(16)-C(19)-H(19A)	109.5
C(16)-C(19)-H(19B)	109.5
H(19A)-C(19)-H(19B)	109.5
C(16)-C(19)-H(19C)	109.5
H(19A)-C(19)-H(19C)	109.5
H(19B)-C(19)-H(19C)	109.5

Table S5. Selected hydrogen bonds for **5** [Å and °].

D–H···A	d(D–H)	d(H···A)	d(D···A)	<(DHA)
O(1)-H(1A)...N(6)#1	0.84	2.09	2.8754(15)	155.4
C(12)-H(12A)...O(2)	0.98	2.55	3.1108(18)	116.4
C(13)-H(13B)...O(2)#2	0.98	2.55	3.4507(19)	153.2
C(14)-H(14A)...O(2)	0.98	2.35	2.8981(17)	114.8
N(6)-H(6D)...N(2)#3	0.898(18)	2.307(18)	3.1045(16)	147.9(14)
N(5)-H(5D)...N(4)	0.857(17)	2.262(16)	2.8409(17)	124.9(13)
N(5)-H(5C)...N(2)#4	0.908(18)	2.199(18)	3.0411(17)	154.0(14)
N(5)-H(5C)...O(3)#4	0.908(18)	2.645(17)	3.2768(15)	127.4(12)
N(6)-H(6C)...O(2)	0.881(17)	2.115(16)	2.7273(16)	126.0(14)

Symmetry transformations used to generate equivalent atoms:
#1 -x+2,-y+2,-z+1; #2 x-1,y,z; #3 x+1,y,z; #4 -x,-y+1,-z+1

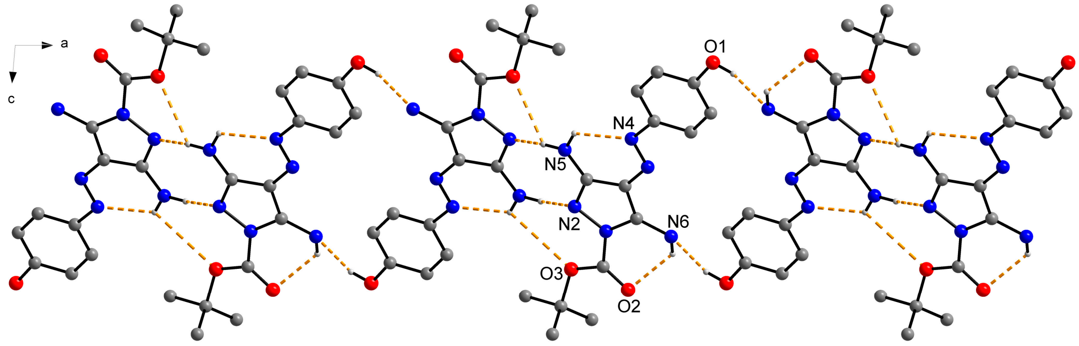

Figure S1. A part of the crystal structure of **5** showing the formation of supramolecular 1*D* chain and selected N–H···N, N–H···O and O–H···N hydrogen bonds (dashed lines)

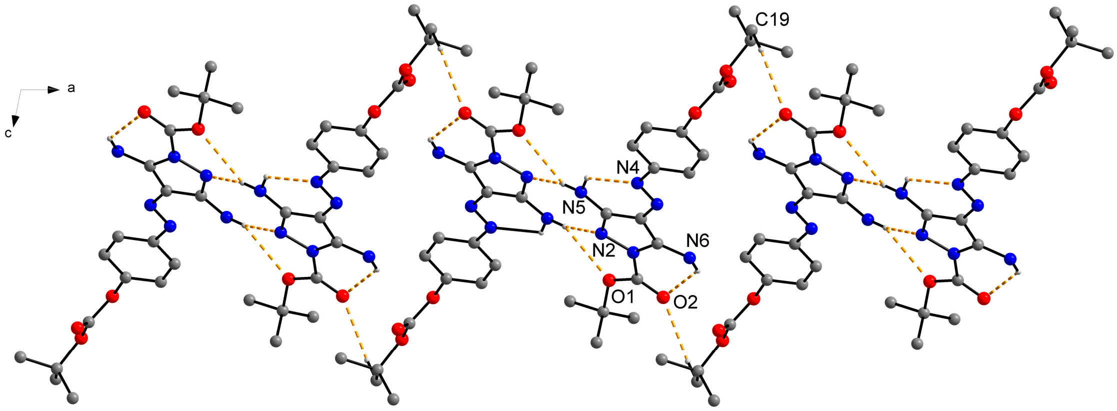

Figure S2. A part of the crystal structure of **3** showing the formation of supramolecular 1*D* chain and selected N–H···N, N–H···O and C–H···O hydrogen bonds (dashed lines)

Table S6. Selected hydrogen bonds for **3** [Å and °].

D–H···A	d(D–H)	d(H···A)	d(D···A)	<(DHA)
C(12)-H(12A)...O(2)	0.98	2.57	3.123(2)	115.9
C(14)-H(14B)...N(4)#1	0.98	2.67	3.628(2)	166.8
C(17)-H(17B)...O(4)	0.98	2.40	2.991(2)	118.1
C(18)-H(18A)...O(4)	0.98	2.45	2.998(2)	115.3
C(19)-H(19C)...O(2)#2	0.98	2.65	3.571(2)	156.6
N(5)-H(5B)...O(1)#3	0.90(2)	2.66(2)	3.3269(19)	131.9(17)
N(5)-H(5B)...N(2)#3	0.90(2)	2.13(2)	2.997(2)	163.4(19)
N(6)-H(6B)...O(2)	0.88(2)	2.17(2)	2.744(2)	122.9(16)
N(5)-H(5A)...N(4)	0.87(2)	2.25(2)	2.842(2)	124.5(15)
N(6)-H(6A)...N(5)#4	0.88(2)	2.45(2)	3.151(2)	136.9(17)

Symmetry transformations used to generate equivalent atoms:
#1 -x+1,-y,-z+1; #2 -x+2,-y+1,-z+1; #3 -x,-y,-z+1; #4 x+1,y,z

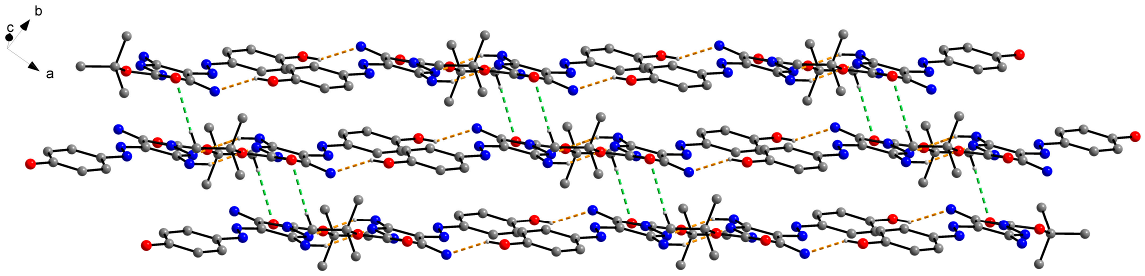

Figure S3. A part of the crystal structure of **5** showing the formation of supramolecular layers and selected C–H···O non-covalent contacts (dashed lines)

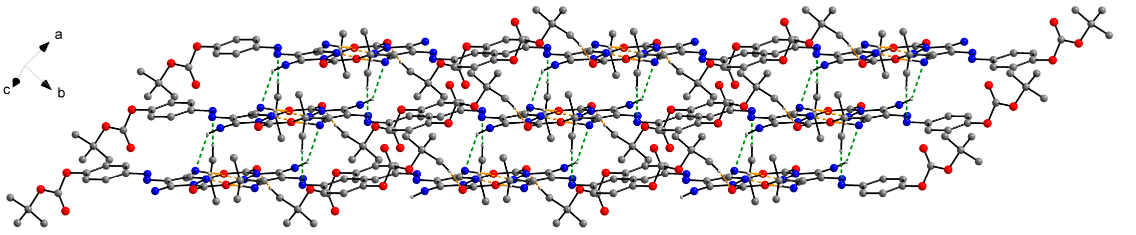

Figure S4. A part of the crystal structure of **3** showing the formation of supramolecular layers and selected C–H···N and N–H···N non-covalent contacts (dashed lines)
